# Lithium titanate hydrates with superfast and stable cycling in lithium ion batteries

**DOI:** 10.1038/s41467-017-00574-9

**Published:** 2017-09-20

**Authors:** Shitong Wang, Wei Quan, Zhi Zhu, Yong Yang, Qi Liu, Yang Ren, Xiaoyi Zhang, Rui Xu, Ye Hong, Zhongtai Zhang, Khalil Amine, Zilong Tang, Jun Lu, Ju Li

**Affiliations:** 1 State Key Lab of New Ceramics and Fine Processing, School of Materials Science and Engineering, Tsinghua University, Beijing, 100084 China; 2 Department of Nuclear Science and Engineering, Massachusetts Institute of Technology, Cambridge, Massachusetts 02139 USA; 3 Department of Chemistry, Tsinghua University, Beijing, 100084 China; 4 X-ray Science Division, Advanced Photon Source, Argonne National Laboratory, Lemont, Illinois 60439 USA; 5 Chemical Sciences and Engineering Division, Argonne National Laboratory, Lemont, Illinois 60439 USA; 6 Department of Materials Science and Engineering, Massachusetts Institute of Technology, Cambridge, Massachusetts 02139 USA

## Abstract

Lithium titanate and titanium dioxide are two best-known high-performance electrodes that can cycle around 10,000 times in aprotic lithium ion electrolytes. Here we show there exists more lithium titanate hydrates with superfast and stable cycling. That is, water promotes structural diversity and nanostructuring of compounds, but does not necessarily degrade electrochemical cycling stability or performance in aprotic electrolytes. As a lithium ion battery anode, our multi-phase lithium titanate hydrates show a specific capacity of about 130 mA h g^−1^ at ~35 C (fully charged within ~100 s) and sustain more than 10,000 cycles with capacity fade of only 0.001% per cycle. In situ synchrotron diffraction reveals no 2-phase transformations, but a single solid-solution behavior during battery cycling. So instead of just a nanostructured intermediate to be calcined, lithium titanate hydrates can be the desirable final destination.

## Abstract (web summary)

Water is usually not favorable in high-voltage window aprotic electrolytes. Here the authors discover some lithium titanate hydrates that allow superior power rate and ultralong cycle life in aprotic electrolytes.

## Introduction

**Fig. 1:**
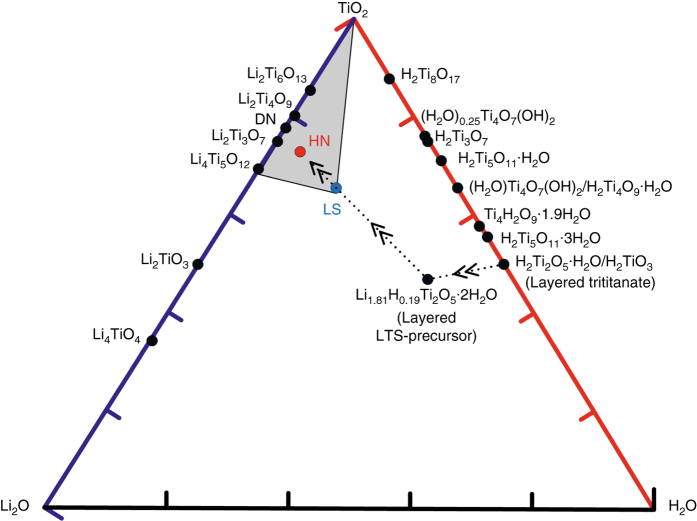
Calculated ternary phase diagram of Li_2_O–TiO_2_–H_2_O ternary composition space. The *arrow* demonstrates the ODIN process, the *black dot* shows the common phase in the Li_2_O–TiO_2_–H_2_O ternary composition space; and the *blue dot* is the new phase LS; the *shadow* region illustrate the new mixed phase HN composed of Li_4_Ti_5_O_12_–TiO_2_–LS. See also Supplementary Movie 1

Fast-charging electronic devices have experienced dramatic development recently^1^. Compounds on the binary Li_2_O–TiO_2_ composition line, such as Li_4_Ti_5_O_12_ (2Li_2_O•5TiO_2_, LTO) and various TiO_2_ polymorphs (TO), are generally considered the most promising anode materials for Li-ion batteries in terms of rate capability and cycling stability, as well as the improved safety over graphite anode^2–6^. Producing nanostructured materials on the Li_2_O–TiO_2_ composition line often requires water-based synthesis such as hydrothermal or sol-gel approaches, and thus one often deals with reaction intermediates that contain water (lithium titanate hydrates, LTHs) in the Li_2_O–TiO_2_–H_2_O ternary composition space (Fig. 1). Because water is considered “harmful” in high-voltage window aprotic electrolytes (free water can be highly reactive to LiPF_6_, lithium metal anode and lithium alkyl carbonates)^7^, most researchers calcine the nanostructured LTHs to completely remove all water by raising temperature to above 500 °C^8^. However, this can cause an unwanted side effect of coarsening and aggregation of the structure. Herein, we demonstrate that the high-temperature calcining may not be necessary. One may only need to remove the more loosely bound water (such as adsorbed and crystallographic water) by heating to a much lower temperature of <260 °C, which does not induce significant coarsening of the nanostructure. The deeply trapped water inside LTHs, or pseudohydrates (i.e., hydroxide or hydroxonium ions or as –OH and –H groups)^9, 10^, does not necessarily degrade stability or performance in aprotic electrolytes, even with H_2_O: TiO_2_ molar ratio as high as 0.41. Indeed, the trapped water can promote structural diversity and nanostructuring that could be highly beneficial for battery performance in aprotic electrolytes.

**Fig. 2:**
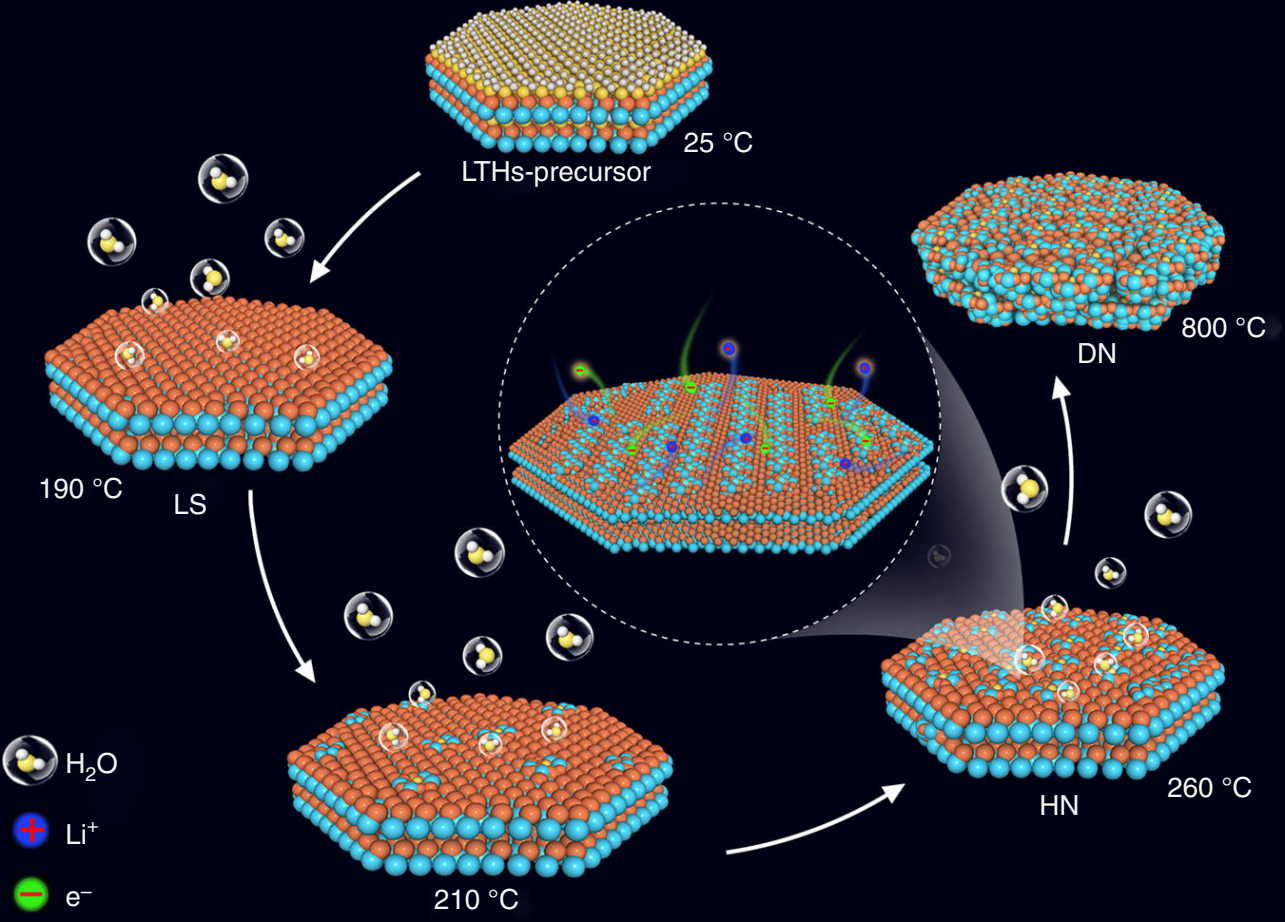
Schematic diagram in the dehydration process and the fast lithium insertion/extraction within the hydrated nanocomposite (HN) material in battery. The tiny clusters appeared on the nanosheet after 190 °C represent LTO and TO nanocrystallites, and the clusters grow gradually to bigger crystals with the increase of temperature

In this paper, a series of novel materials in the Li_2_O–TiO_2_–H_2_O ternary composition space are discovered via an optimized dehydration induced nanostructuring (ODIN) approach, which is illustrated in Fig. 2 with an accompanying animation (Supplementary Movie 2). They show better electrochemical performances compared to all the Li_2_O–TiO_2_ materials reported so far (see the comparison in Supplementary Table 1), including those after nanostructuring, doping and/or coating. As lithium ion battery anode, our novel lithium titanate hydrates can still show a specific capacity of about 130 mA h g^−1^ at ~35 C (fully charged within ~100 s) and sustain more than 10,000 cycles with capacity fade of only 0.001% per cycle. This discovery indicates that the level of dehydration after hydrothermal or sol-gel synthesis should be carefully optimized, since phase transformation driven by dehydration could be a valuable tool for creating composites with nanostructure refinement, and mixed battery and pseudocapacitor features^11^, whereas complete dehydration by further heating can coarsen the structures and actually degrade electrochemical performances.

## Results

### Synthesis and characterization of lithium titanate hydrates

**Fig. 3:**
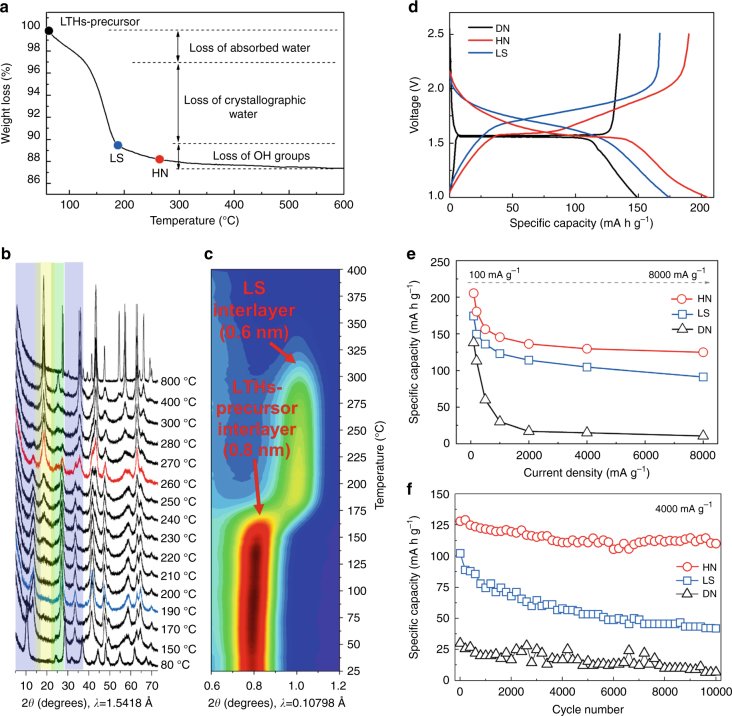
Characterization of as synthesized materials in the Li_2_O–TiO_2_–H_2_O ternary space. **a** TG analysis; **b** Ex situ XRD patterns and (**c**) contour plot of in situ HEXRD profile (*red* represents a high intensity and *blue* represents a low intensity) of LTHs-precursor heated to different temperatures. Comparison among LS, HN and DN electrode materials in the following three cases: **d** Charge–discharge profiles at 100 mA g^−1^ between 1.0 and 2.5 V (vs Li/Li^+^); **e** Rate capabilities at different current densities from 200 to 8000 mA g^−1^ and **f** cycling stability at 4000 mA g^−1^. All RT electrochemical measurements **d**–**f** were carried out in two-electrode 2032 coin-type half-cells

Two-dimensional (2D) crystals may provide kinetic advantage due to plenty of fast Li^+^ conducting pathways^12^. We set out with layered hydrogen trititanates with a formula of H_2_Ti_*n*_O_*2n+*1_·H_2_O (or Na_*x*_H_2-*x*_Ti_*n*_O_2*n*+1_·H_2_O, 2 ≤ *n* ≤ 9, 0 ≤ *x* ≤ 2, due to the incomplete ionic exchange reaction), such as H_2_Ti_2_O_5_·H_2_O (2H_2_O•2TiO_2_) and H_2_Ti_3_O_7_ (H_2_O•3TiO_2_), which are well known to form nanotubes and nanosheets^8^ but unable to support long-life electrochemical cycling in aprotic electrolyte. We then produced a 2D Li_1.81_H_0.19_Ti_2_O_5_·2H_2_O precursor (hereafter referred to as LTHs-precursor) by hydrothermal lithiation of layered hydrogen trititanates in LiOH solution^13^, which preserves the layered nature with interlayer spacing of 0.8 nm (Supplementary Fig. 1). We then systematically study their performance in electrochemical cycling, as we gradually dehydrate the material (see the *arrow* direction in Fig. 1 and Supplementary Movie 1) by raising the heating temperature. TG (DSC) curves (Fig. 3a and Supplementary Fig. 2) and ex situ XRD patterns (Fig. 3b) reveal that the sample underwent three different stages in the ODIN process. The first weight loss region was 50–130 °C, which corresponded to the loss of absorbed water, and the second region was 130–190 °C, which was mainly attributed to the loss of crystallographic water, with LTHs-precursor transforming into a new layered structure, hereon called LS. In situ high-energy X-ray diffraction (HEXRD, Fig. 3c) technique illustrated the transformation of the layered structure. During the thermal ramping, the reflection of layered structure (200) for LTHs-precursor maintained until ~180 °C, and then shifted to a higher 2*θ* angle at above 190 °C, leading to the decrease of interlayer spacing to 0.6 nm (Supplementary Fig. 3). Fourier transform infrared spectroscopy (FTIR) demonstrates that the main state of proton in LS is hydrogen-bonded Ti–OH (Supplementary Fig. 4). A possible chemical formula of LS is Li_1.25_H_1.63_Ti_2_O_5.44-*σ*_ (*σ* represents the O vacancy), based on inductively coupled plasma mass spectroscopy (ICP-MS) and elemental analyzer measurements. Prior to the formation of LS (before dehydrating to 190 °C), the electrochemical performance of dehydrated LTHs-precursor is not satisfactory, because the “free water” can be driven out of the material during electrochemical cycling and therefore severely degrade the aprotic electrochemical system (Supplementary Fig. 5) as previously understood^7^.

The last weight loss region was 190–400 °C, which corresponded to the gradual collapse of the layered structure and the growth of three-dimensional (3D) LTO and TO nanocrystallites via a topotactic transformation reaction^14, 15^. With rising temperature in this regime, an increasing proportion of Li_4_Ti_5_O_12_ and anatase TiO_2_ nanocrystallites was obtained (as shown in the *yellow* region and *green* region in Fig. 3b, respectively); correspondingly, the proportion of LS decreased (as shown in the *purple* region in Fig. 3b), and the material became more defective due to loss of water in the 2D lattice. Notably, the peak corresponding to the interlayer spacing of LS disappeared above 350 °C^8^, implying the collapse of layered structure. As the temperature reached 400 °C, the diffraction peaks of Li_4_Ti_5_O_12_ and TiO_2_ became steep, indicating growth of 3D crystals. To make our point of pseudohydrates being benign for room-temperature (RT) electrochemical performance, we pick a final dehydration temperature of 260 °C, where a nanocomposite material consisting of defective LS, spinel Li_4_Ti_5_O_12_ (JCPDS No. 49-0207) and anatase TiO_2_ (JCPDS No. 89-4921) nanocrystallites was obtained, with the individual sets of planes indexed (Supplementary Fig. 6). We call this material the hydrated nanocomposite (HN) state, which still contains significant amount of water in its defected LS component to keep the layered structure, albeit less than LS as illustrated in Fig. 1. The chemical formula of HN is Li_1.39_H_1.18_Ti_2_O_5.29-*σ*_ (*σ* represents the O vacancy) based on the same measurements for LS. It is important to note that the hydrogen atoms are distributed homogeneously throughout the bulk of the nanosheets from time-of-flight secondary ion mass spectrometry (TOF-SIMS) depth profile (Supplementary Fig. 7), distinct from surface hydrogenation^16^.

Beyond 400 °C, no more water came out. The final product at 800 °C is Li_4_Ti_5_O_12_ and rutile TiO_2_ (JCPDS No. 21-1276), as anatase TiO_2_ transformed to rutile TiO_2_ at a relatively high temperature of 800 °C. This completely water-free Li_4_Ti_5_O_12_-TiO_2_ nanocomposite (hereafter referred to as DN, dry nanocomposite) is also measured for RT electrochemical performances for comparison. Note that DN (which is the route most researchers took to obtain electrodes on Li_2_O–TiO_2_ composition axis after hydrothermal synthesis) has coarser structures than HN, due to coarsening and aggregation at higher temperatures (Supplementary Figs. 8, 10). The specific surface area decrease caused by the coarsening is also characterized by N_2_ adsorption/desorption (Supplementary Fig. 11). This tradeoff between “water removal” and “nanostructure coarsening” and its impact on electrochemical performance is at the heart of the current work. We will show that removing all water is often an overkill. Instead, the as-synthesized LS and HN through ODIN give the best results.

### Electrochemical behavior of lithium titanate hydrates

Electrochemical performance of hydrated LS and HN materials were evaluated and compared to water-free DN. Figure 3d provides the galvanostatic discharge/charge curves of HN, LS and DN at a current density of 100 mA g^−1^ between 1.0 and 2.5 V. LS has a discharge capacity of 174 mA h g^−1^ and the nearly constant slope of current-potential curve indicates a quasi-solid-solution behavior. On the other hand, DN reveals a typical battery type of discharge curve with a well-defined voltage plateau and delivers 149 mA h g^−1^ discharge capacity. HN, with a discharge storage behavior combining the features of the following three: sloping current-potential for LS, nanosized LTO and TO^17, 18^, as well as the additional Li storage at the interfaces, exhibiting high capacity of 205 mA h g^−1^. Cyclic voltammetry (CV) curves for LS, HN and DN materials further demonstrate the composite nature of HN electrode (Supplementary Fig. 12).

The rate capability of the three materials is shown in Fig. 3e. When discharged at 100 mA g^−1^, owing to the surface reactions of LS together with the additional Li storage at the interfaces among Li_4_Ti_5_O_12_-TiO_2_-LS, HN delivers a reversible capacity of 205 mA h g^−1^, which is higher than LS (174 mA h g^−1^) or DN (138 mA h g^−1^). As the current density increases to 8000 mA g^−1^, the capacity of HN and LS gently decrease to 124 mA h g^−1^ and 91 mA h g^−1^, respectively, which demonstrate their superior rate capabilities. The capacity of DN, in contrast, plummets to 17 mA h g^−1^ at 2000 mA g^−1^ and ends in almost zero at higher current density. As a result, LS demonstrates a quite stable performance for ultrafast lithium ions insertion/extraction at a high current rate of 8000 mA g^−1^ (~70 C) for 1000 cycles with 76% capacity retention of its initial capacity 106 mA h g^−1^ (Supplementary Fig. 13). The HN electrode exhibits superior cycling capacity of about 130 mA h g^−1^ at 4000 mA g^−1^ (fully charged within ~100 s) and sustains >10,000 cycles with 86% capacity retention (Fig. 3f). Moreover, nearly complete nanosheet morphology of HN was retained after 10,000 ultrafast cycling under transmission electron microscopy (TEM; Supplementary Fig. 14) observation. From X-ray photoelectron spectroscopy (XPS) analysis of the electrodes after cycling (Supplementary Fig. 15), we know that Li_2_CO_3_ and ROCOOLi species are the major components on the surface of LS and HN electrodes, due to the interfacial reactions between the electrodes and electrolyte, but with little influence on their performance^19^. The high rate capacity of DN at 4000 mA g^−1^, in contrast, drops rapidly to < 20 mA h g^−1^ for the first 500 cycles, and failed completely after 1000 cycles. In terms of mass loading, rate and cyclability (see comparison with literatures in Supplementary Table 1), the HN and LS are two very promising materials (both hydrated) for fast-charging Li-ion batteries or even supercapacitors.

**Fig. 4:**
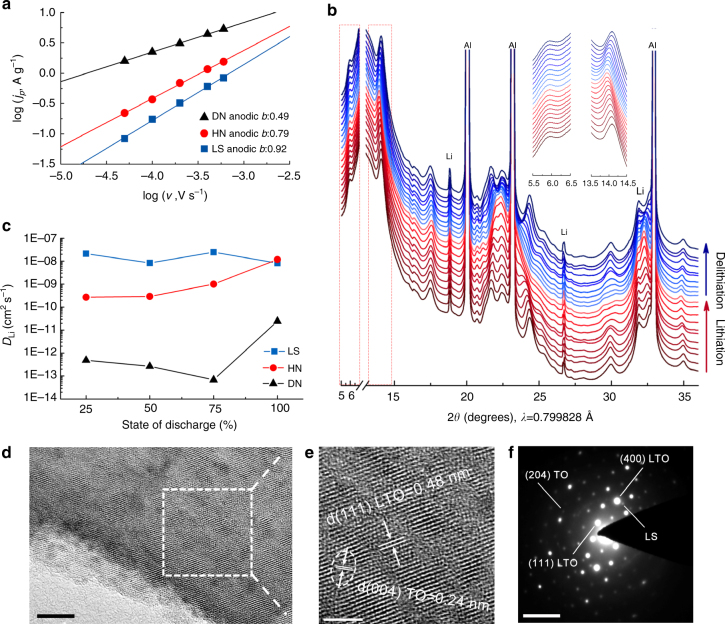
Illustration for electrochemical performance enhancement mechanism of lithium titanate hydrates. **a**, *b*-values determination of anodic peaks with sweep rate from 0.05 mV s^−1^ to 0.6 mV s^−1^ for LS, HN and DN electrodes; **b** in situ synchrotron XRD results during the third cycle of LS electrode cycled at 100 mA g^−1^, some main peaks of LS at 6° and 14° are highlighted in the insert; **c** Li-ion diffusion coefficient at various state of discharge; **d** HRTEM image of a HN nanosheet (*scale bar*, 10 nm) and **e** Magnified image of the selected region in **d** with a spacing of 0.48 nm of Li_4_Ti_5_O_12_ and 0.24 nm of TiO_2_ (*scale bar*, 5 nm); **f** SAED pattern of HN (*scale bar*, 5 1/nm)

The charge storage mechanism was characterized by sweep voltammetry (Supplementary Fig. 18), assuming that the current obeys a power-law relationship with the sweep rate (Fig. 4a):^11, 12^

1 $$i{\rm{ = }}a{v^b}$$

where *a* and *b* are adjustable parameters. For LS, *b*-value is 0.92, close to 1, indicating that the electrochemical kinetics is mainly generalized surface-controlled redox reaction. The *b*-value of DN, on the other hand, approaches a value of 0.5 (diffusion-controlled redox reaction). HN, as a combination of high-rate feature for faradic pseudocapacitance (LS) and high-capacity feature for diffusion-controlled process (Li_4_Ti_5_O_12_ and TiO_2_), presents an intermediate *b*-value of 0.79, and leads to the best capacity/cyclability combination (Supplementary Table 1).

The structural transformation of LS at different stages of extraction-insertion was examined via in situ synchrotron diffraction (Fig. 4b) at Argonne’s Advanced Photon Source, demonstrating a solid-solution behavior^20^ over a large concentration range. No two-phase transformation was detected, which involves changes of peak heights at distinct locations. The lattice constant (peak position) is seen to change linearly and reversibly with the state of charge. The change of interlayer spacing corresponding to ~6° is quite small, implying a fast transport kinetics of Li^+^ through the layered structure. It is known that the solid solution behavior could emerge when increasing the (dis)charge rate or decreasing the particle size^20, 21^. Consequently, the fact that solid solution behavior appeared at relatively low current density (<0.5C) suggests that the electrode may possess good potential in achieving super high-rate capability^12, 22, 23^. The Li-ion diffusion coefficients (*D*_Li_) of the three electrodes at different discharge depths are shown in Fig. 4c (see more details in Supplementary Table 4 and Supplementary Figs. 20, 22). The Li-ion diffusivity in LS (8.21 × 10^−9^−2.46 × 10^−8^ cm^2^ s^−1^) is about 3–5 orders of magnitude higher than that in DN (6.81 × 10^−14^−2.46 × 10^−11^ cm^2^ s^−1^). This tremendous difference is attributed to the nanostructure coarsening of DN and 2D layered structure of LS, which makes LS a fast ion conductor. Just as shown in the animation and Fig. 4d, e, the HN was composed of uniformly dispersed Li_4_Ti_5_O_12_ and TiO_2_ crystallites about 2–5 nm on a LS nanosheet. Besides, the thickness of the HN nanosheets is only about 5 nm via atomic force microscope (AFM) analysis (Supplementary Fig. 23). Lattice fringes of anatase TiO_2_ (004) plane and Li_4_Ti_5_O_12_ (111) plane with a 40° angle are observed in Fig. 3d. Similarly, lattice fringes of anatase TiO_2_ (004) plane and LS plane with a 28° angle (Supplementary Fig. 24) are also discovered. In selected-area electron diffraction pattern (SAED, Fig. 4f), the HN composite material was constituted by a set of sharp spots corresponding to (111), (311) and (400) planes of Li_4_Ti_5_O_12_, some weak spots corresponding to (204) planes of anatase TiO_2_ and unknown planes of LS, proving that it is a combination of LS and Li_4_Ti_5_O_12_ monocrystals with minor amount of anatase TiO_2_ monocrystals. As ion conductivity can be greatly enhanced by the space charge and percolation^24^, hybrid nanostructured HN can exhibit outstanding Li-ion diffusion coefficients (2.72 × 10^−10^−1.17 × 10^−8^ cm^2^ s^−1^).

HN composite (consisting of LS/LTO/TO mixture) during battery cycling was also examined using in situ synchrotron diffraction. In Supplementary Fig. 25, the diffraction peaks of the LS component show a continuous shift in small range during the lithiation/delithiation process, which demonstrates a solid-solution behavior. Interestingly, no obvious two-phase transitions were found in the in situ XRD pattern of the LTO and TO components either. It is well known that the redox mechanism of bulk LTO and TO involves two-phase transformations^17, 18, 25^. However, when the particle size decreases to several nanometers (<5 nm), many electrode materials with two-phase behavior will change to solid-solution behavior, showing a single solid-solution (such as regular solution model with nonzero enthalpy of mixing) phase field with large solubility range for Li^26, 27^. This would allow skipping the nucleation-and-growth kinetics of two-phase transformations, which involves an interfacial energy plus surface energy penalty that could be too expensive and slow for small particles undergoing fast changes. Here, the capacitor-like behavior indeed demonstrates the nano-size effect of our HN nanocomposite material as well as the advantages of ODIN strategy, which leads to the best electrode with super high-rate capability.

ODIN is a simple approach to obtain sub-10 nm LTO/TO crystallites firmly anchored on LS. While nanocrystallites could more easily accommodate the structural changes during the ultrafast insertion and extraction process for the cycling stability improvement^28^, a major problem is the aggregation of nanocrystallites during preparation and battery cycling^29^. The LS substrate plays a crucial role in structural anchoring to restrict the growth and prevent the aggregation of LTO and TO nanograins during RT battery cycling, thus making the ultrastable performance of HN possible. Lattice distortion and disorder in HN composite at the interfaces among LTO-TO-LS (Fig. 4e and Supplementary Fig. 26) and some disordered protons located in the interface layer (detected by ^1^H solid state magic-angle spinning nuclear magnetic resonance (MAS-NMR), Supplementary Fig. 27) may provide more active sites for Li storage and more crystal defects for conductivity improvement^30–32^. In addition, when the size of a crystal decrease to several nanometers, not only is the diffusion distance of lithium ions shortened^33^, the local material properties could even change as well^34, 35^, which might greatly enhance the ionic and electrical conductivity of the crystal.

**Fig. 5:**
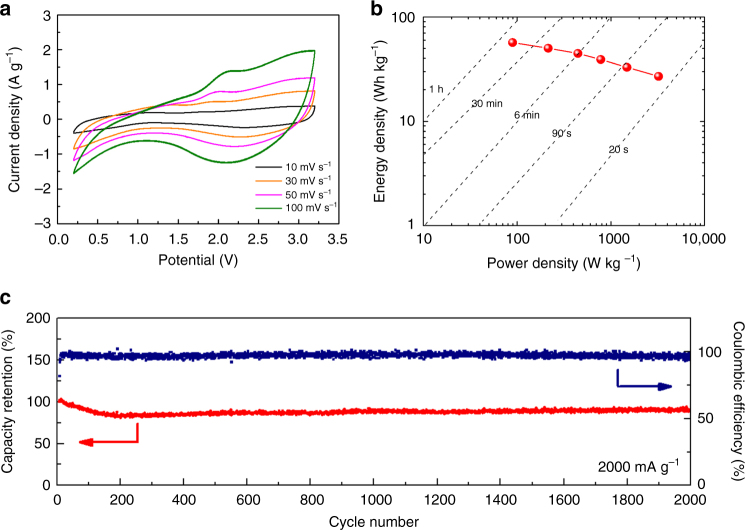
Electrochemical performance of hybrid supercapacitor using an activated carbon (AC) and hydrated nanocomposite (HN) composite. **a**, CV curves in various scan rates from 10 to 100 mV s^−1^; **b**, Ragone plots of power density vs. energy density, based on the total mass of active materials in both electrodes; **c**, Cycle stability and Coulombic efficiency at a current density of 2000 mA g^−1^

To demonstrate the practical utility of HN anode, we tested the electrochemical performance of hybrid supercapacitor using the commercial activated carbon (AC, YP-50F by Kuraray Chemical Co., LTD) and HN composite at an optimal mass ratio of *m*_positive_/*m*_negative_ = 3:1, between 0.2 and 3.2 V. Different from the symmetric supercapacitor, the AC//HN hybrid supercapacitor exhibits a gradual deviation from the ideal rectangular shape with increasing scan rate (Fig. 5a), owing to the overlapping of two different energy-storage mechanisms. The Ragone plot (Fig. 5b), which exhibits the trade-off between energy and power densities in the hybrid supercapacitor, was calculated from the cyclic voltammograms using the equations of Zhang et al^36^. (the energy density and power density were based on the total mass of active materials in both electrodes). The hybrid supercapacitor not only could deliver up to 57 Wh kg^−1^ at relatively low-power density (90 W kg^−1^), but also could maintain 27 Wh kg^−1^ with an extremely high-power density of about 3200 W kg^−1^ (fully charged within 30 s). It also exhibited an ultrastable cycling performance (2000 cycles with capacity fade of only 5 × 10^−3^ % per cycle) under a current density of 2000 mA g^−1^ (Fig. 5c), proving its ultrafast transport of both e^−^ and Li^+^ in aprotic electrolyte systems (Supplementary Movie 3). In addition, we have also assembled full batteries with LiFePO_4_ cathode vs. HN anode (Supplementary Fig. 28), with tolerable gassing. The excellent cyclability of the full cell validates LTHs as promising electrode materials for the real-life fast-charging electronics and electric vehicles.

## Discussion

In summary, we have discovered a series of novel lithium titanate hydrates in the Li_2_O–TiO_2_–H_2_O ternary composition space. The LTHs exhibit outstanding high-rate performance, and even more intriguingly, extraordinary RT cycling stability. Once we realize that not all water is bad, and some water can actually do good, it might be possible to synthesize many more electrode materials with superior power density and ultralong cycle life in the Li_2_O–TiO_2_–H_2_O composition space, that beat what are available on the Li_2_O–TiO_2_ binary axis (the “dry” side), by taking advantages of the structural diversity of hydrated crystals (2D layered), and dehydration induced phase transformation and nanostructure refinement.

The ODIN approach may be generalized to other hydrated compound composition spaces as well. The materials design philosophy we have established is the following: (i) water may not be bad actor for RT electrochemical cycling in aprotic electrolyte, if they are trapped in the lattice and not free (our empirical evidence is that the TG desorption temperature should be above 190 ºC or so); (ii) as water promotes structural diversity in hydrothermal or sol-gel process, it could be used to tailor the initial structures such as 2D sheets or nanotubes, which greatly improves the ion diffusivity; (iii) the dehydration process thereafter should be carefully optimized: first, all the “free” water (adsorbed, crystallographic) should be driven out; but as it is not integral part of the lattice, this usually does not cause drastic phase change. Then as temperature is further raised, some pseudohydrates embedded in the lattice are also partially driven out; as they were strongly embedded in the lattice, their partial removal can cause drastic phase transformation. This is actually a golden opportunity to refine the nanostructures, as the old structure is being dismantled and turned into a defected form, and new phases are nucleated as nanocrystals, which must start out very small by definition. These sub-10 nm new phases are well-anchored on the 2D substrate, providing structural stability during high-rate RT cycling, as well as a hybrid supercapacitor-battery kinetics and the best capacity/rate/cyclability combination. Because of this, we believe in many occasions after hydrothermal or sol-gel processing, there could exist an optimal dehydration temperature *T*_optimal_ for achieving the best RT electrochemical performance. Further drying the system above *T*_optimal_ will be counter-productive, since the nanostructures could (i) aggregate and coarsen, (ii) or change from 2D to 3D forms, as we showed for the case of DN, where not only the rate capability degrades, but also the cyclability. As ubiquitous water is in nature and in chemical synthesis, we believe the ODIN synthetic approach could provide a window of opportunity for finding new high-performance electrode materials in aprotic electrolyte systems.

## Methods

### Materials synthesis

A typical preparation procedure of lithium titanate hydrates consists of three steps. First, layered hydrogen trititanate was prepared via hydrothermal reaction between anatase TiO_2_ powders and concentrated NaOH solution at 150 °C for several hours, followed by ion substitution of Na^+^ with H^+^ in 0.5 M HNO_3_ solution for 3 h. Second, layered LTHs-precursor was obtained by chemical lithiation of hydrogen trititanate in a 0.8 M LiOH solution heated at 150 °C for 12 h in a Teflon-lined stainless steel autoclave. Thirdly, a series of lithium titanate hydrates were synthesized by drying the LTHs-precursor at various temperatures ranging from 80 to 400 °C for 3 h in a vacuum. In this article, LS and HN were obtained at 190 and 260 °C, respectively. For comparison, we chose 800 °C as the synthesis temperature for DN, and kept the other heating experimental variables fixed.

### Materials characterization

Ex situ powder X-ray diffraction (XRD) was recorded on a Bruker D8 Advance with Cu Kα radiation (*λ* = 1.5418 Å). In situ XRD measurements were carried out at 11-ID-C beamline (for the dehydration process of LTHs-precursor and the charge and discharge process of HN) and 11-ID-D beamline (for the charge and discharge process of LS) at Advanced Photon Source, Argonne National Laboratory. Scans were collected in transmission mode using fixed wavelengths of 0.10798/0.117418 and 0.799828 Å for 11-ID-C beamline and 11-ID-D beamline, respectively. The 2D patterns were calibrated and converted to the conventional 1D format (intensity vs. 2 theta) by using the GSAS-II program. The data are plotted and analyzed by using Bachir Aoun’s Ranked Data Analysis program^37^. For the dehydration, the LTHs-precursor was heated up to 400 °C with a heating rate of 2 °C min^−1^ and a spectrum data collection rate of one pattern every 60 s. For the charge and discharge process, a coin-cell with a hole for beam path was assembled using the same electrode and electrolyte mentioned below. Thermogravimetric-differential scanning calorimetry (TG-DSC) analysis was carried out using NETZSCH-STA 449 F3 with a heating rate of 2 °C min^−1^ under a continuous flow of Ar (100 ml min^−1^). Inductively coupled plasma mass spectroscopy (ICP-MS) analysis was carried out using iCP QC (Thermo Fisher Scientific, USA) for lithium and titanium content, and elemental analyzer (EuroEA3000) was used for hydrogen and oxygen content. For Ti analysis, as it is difficult to dissolve in HNO_3_, HF was used as the solvent, and the test was repeated for three times. X-ray photoelectron spectroscopy (XPS) spectra data were obtained using Escalab 250XI system (Thermo Fisher Scientific, US). Nitrogen adsorption–desorption isotherms were obtained using an Automated vapor sorption analyzer (Autosorb-iQ2-MP (Quanta Chrome)) at 77.4 K under vacuum. The specific surface area was calculated by the Brunauer-Emmett-Teller (BET) method. The morphology, size and crystal structure of the as-prepared samples were characterized by MERLIN VP Compact for scanning electron microscope (SEM), Hitachi-HT7700 for transmission emission microscopy (TEM), JEM-2100F for high-resolution transmission electron microscopy (HRTEM) and selected-area electron diffraction (SAED). Tapping mode atomic force microscopy (AFM) images were collected on a multimode atomic force microscope from Veeco Instruments, employing Olympus microcantilevers (resonance frequency, 300 kHz; force constant, 42 N m^−1^). ^1^H solid state magic-angle spinning nuclear magnetic resonance (MAS-NMR) spectra was performed on a Bruker Avance III (400 MHz) spectrometer equipped with a triple channel 2.5 mm probe and adamantine was used as reference. The data were recorded using a pulse sequence with the spinning speed of 10K. The samples were prepared and measured after drying over night at 70 °C in a vacuum. Infrared spectra of the powder samples were measured in diffuse reflectance using a Bruker TENSOR II spectrometer. The samples were measured against a KBr background after purging in inert gas to remove gas-phase water. The depth profiling of LTHs was also analyzed by time-of-flight secondary ion mass spectrometry (TOF-SIMS) using dual ion beams TOF-SIMS (TOF.SIMS 5), in which the Cs^+^ ions are for sputtering and the ${\rm Bi}_1^ +$ ions are for analysis.

### Electrochemical measurements

For the electrochemical measurements, the working electrode was prepared by mixing 80 wt% active material, 10 wt% Super P, and 10 wt% poly(vinylidene fluoride) binder in N-methyl-2-pyrrolidinone solvent. The obtained slurry was cast on 16 μm thickness Al foil or 15 μm thickness Cu foil (when HN was prepared as anode in hybrid supercapacitor and full batteries) via scraper machine and dried at 110 °C in a vacuum oven for 12 h. Then roll squeezer was used to enhance the contact between material and foil, together with increasing the compaction density. The 2032-coin-type cells were assembled in an argon-filled glove-box using pure lithium foil (for half cells) or commercial activated carbon (YP-50F by Kuraray Chemical Co., LTD for hybrid supercapacitors) or commercial LiFePO_4_ (for full cells) as counter electrodes, and a microporous membrane (Celgard 2400, USA) as a separator. 80 μL of 1.0 M LiPF_6_ in a mixture (1:1 volume ratio) of ethylene carbonate (EC) and dimethylcarbonate (DMC) was added as the electrolyte. The mass loading of LTHs anode in this work is ~1.0 mg cm^−2^. In hybrid supercapacitor device, the AC cathode is about triple the mass of anode; and in the full batteries, the LiFePO_4_ cathode is about twice the mass of anode. IM6 (Bas-Zahner, Germany) electrochemical workstation was used for cyclic voltammetry (CV) and also for electrochemical impedance spectroscopy (EIS) from 10 mHz to 100 kHz, with a perturbation of 5 mV applied. For in situ XRD measurements, the home-made coin cell (LS on Al foil) was discharged/charged with a constant current at 100 mA g^−1^. All the cells were tested on a LAND 2001A Cell test system and cycled between 1.0–2.5 V (for half cells), 0.2–3.2 V (for hybrid supercapacitors) and 1.2–2.4 V (for full cells) at room temperature.

### Data availability

The relevant data that support the findings of this study are available from the authors upon reasonable request.

## Electronic supplementary material

Supplementary Movie 1

Supplementary Movie 2

Supplementary Movie Movie 3

Supplementary Information
