# Supplementary material for: Lithium titanate hydrates with superfast and stable cycling in lithium ion batteries
Source: Nat Commun. 2017 Sep 20;8:627. doi: 10.1038/s41467-017-00574-9 (PMC5606990; doi:10.1038/s41467-017-00574-9)
Supplement: Supplementary file 4 — Supplementary Information [file 41467_2017_574_MOESM4_ESM.pdf]

Description of Supplementary Files

File name: Supplementary Information

Description: Supplementary figures, supplementary tables, supplementary notes and supplementary references.

File name: Supplementary Movie 1

Description: Animation of ODIN approach in Li_2O–TiO_2–H_2O ternary composition space

File name: Supplementary Movie 2

Description: Animation of ODIN approach on a nanoscale

File name: Supplementary Movie 3

Description: Animation of lithium titanate hydrates in battery

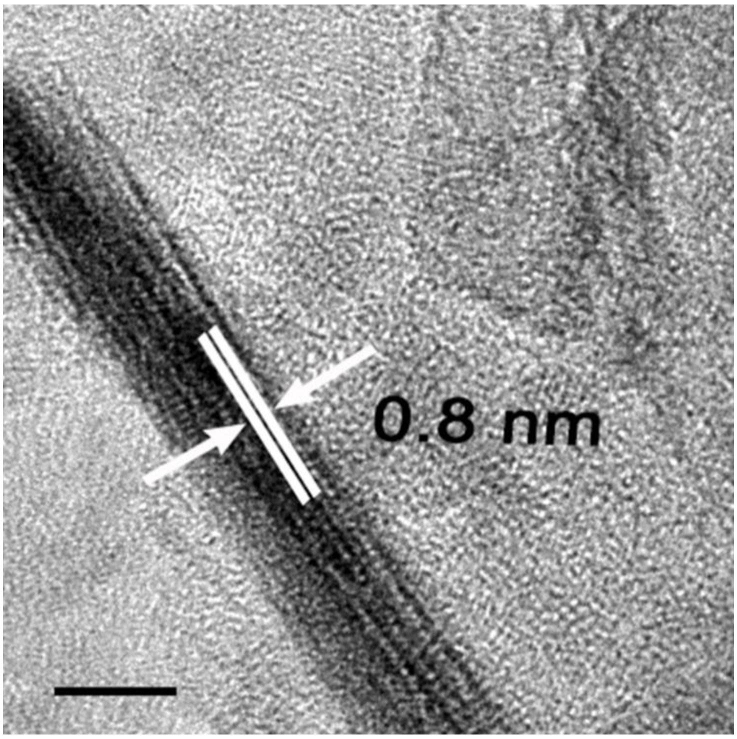

Supplementary Figure 1 | HRTEM image of lithium titanate hydrates precursor (*LTHs-precursor*). *Scale bar*, 5 nm.

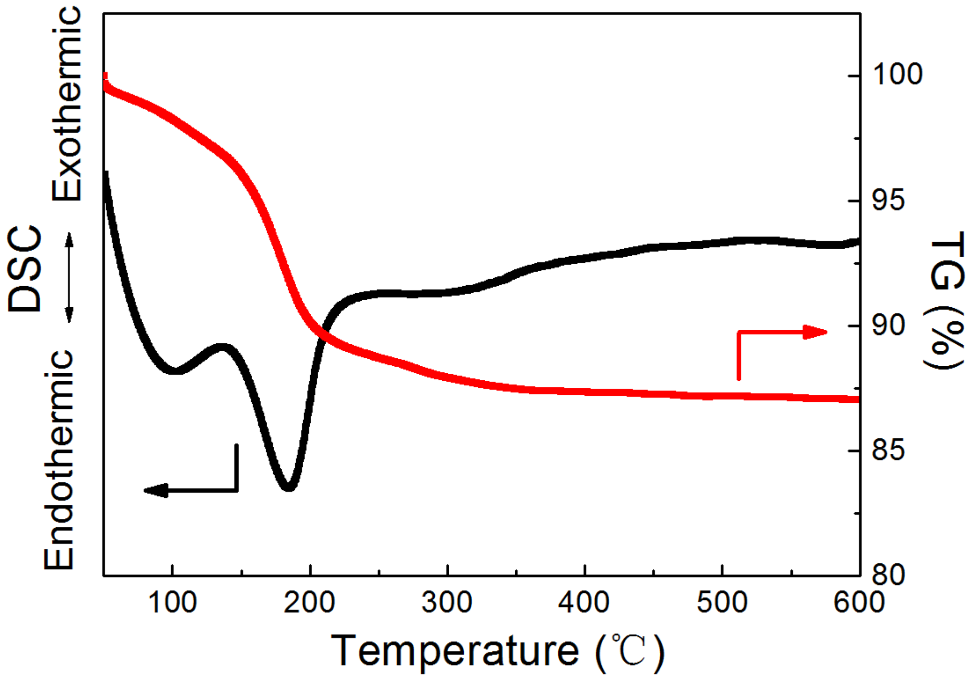

Supplementary Figure 2 | TG-DSC curves of lithium titanate hydrates precursor (*LTHs-precursor*).

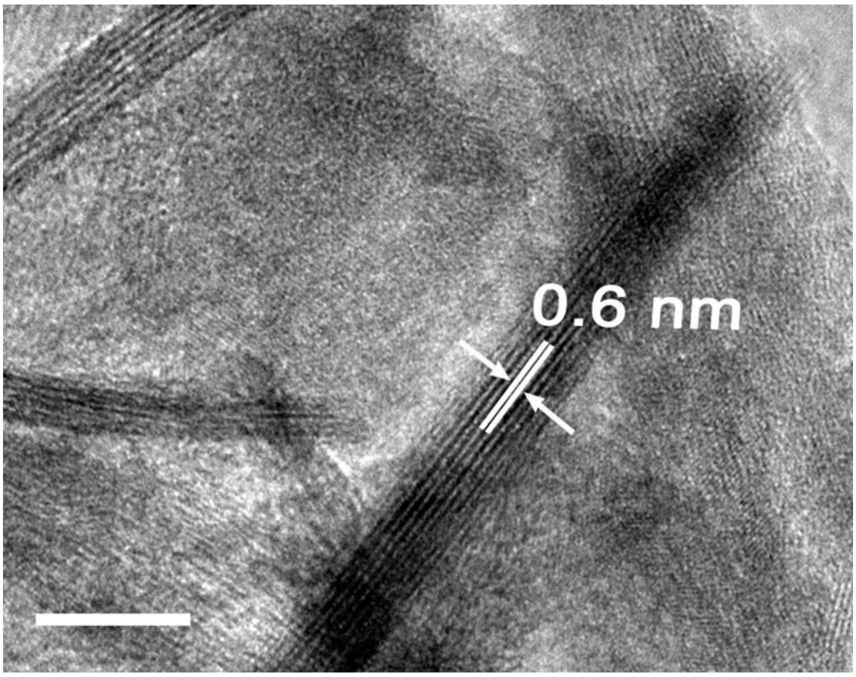

Supplementary Figure 3 | HRTEM image of layered structure (*LS*). *Scale bar*, 10 nm.

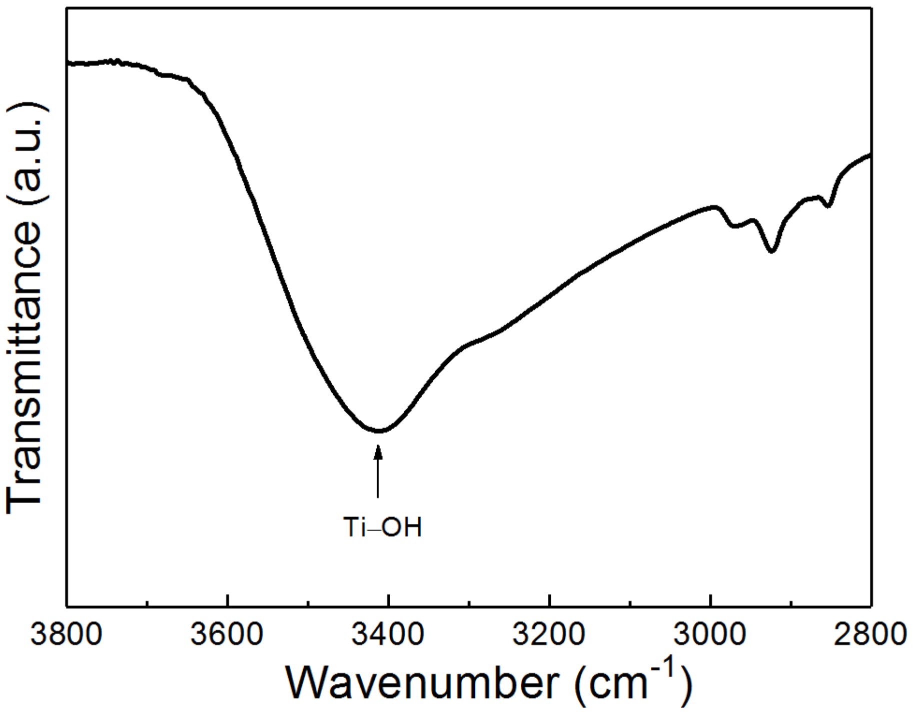

Supplementary Figure 4 | FTIR spectra of layered structure (*LS*).

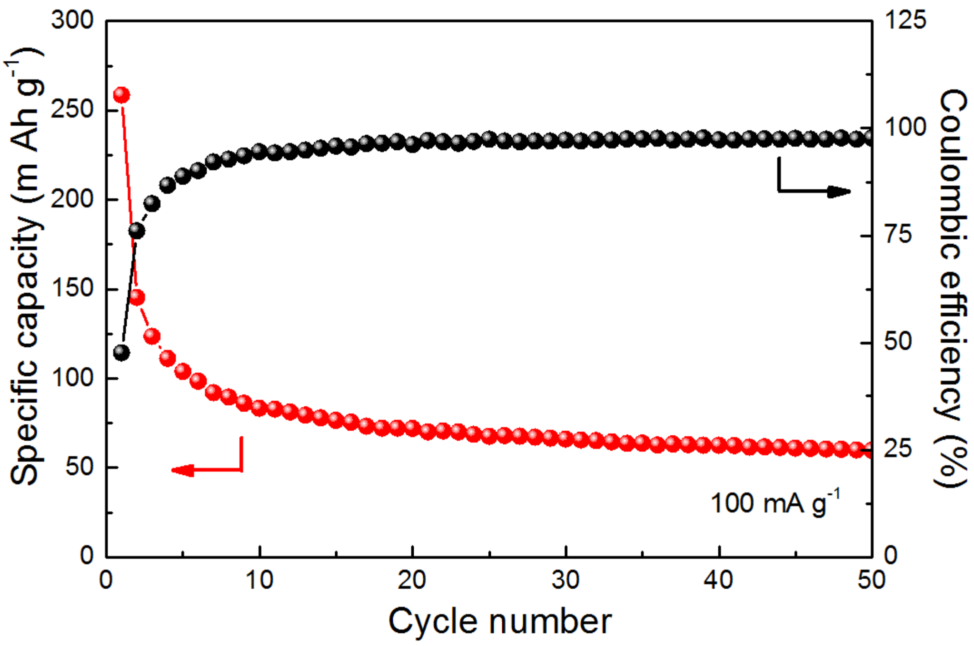

Supplementary Figure 5 | Cycling stability and Coulombic efficiency of lithium titanate hydrates precursor (*LTHs-precursor*) at 100 mA g^{-1}.

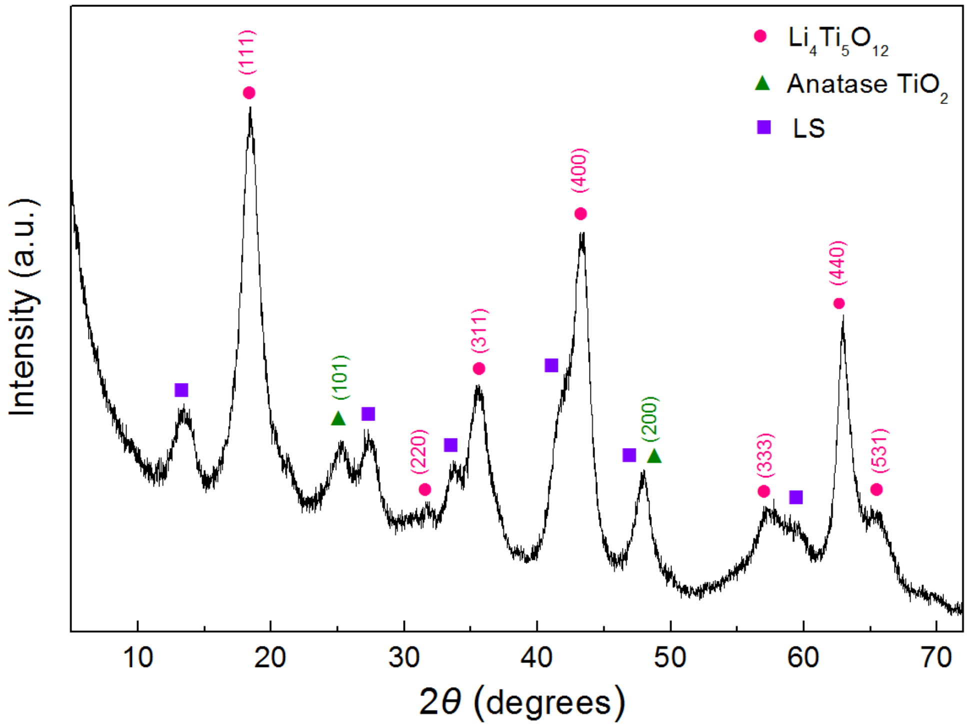

Supplementary Figure 6 | XRD patterns of hydrated nanocomposite (*HN*) with the individual sets of planes indexed.

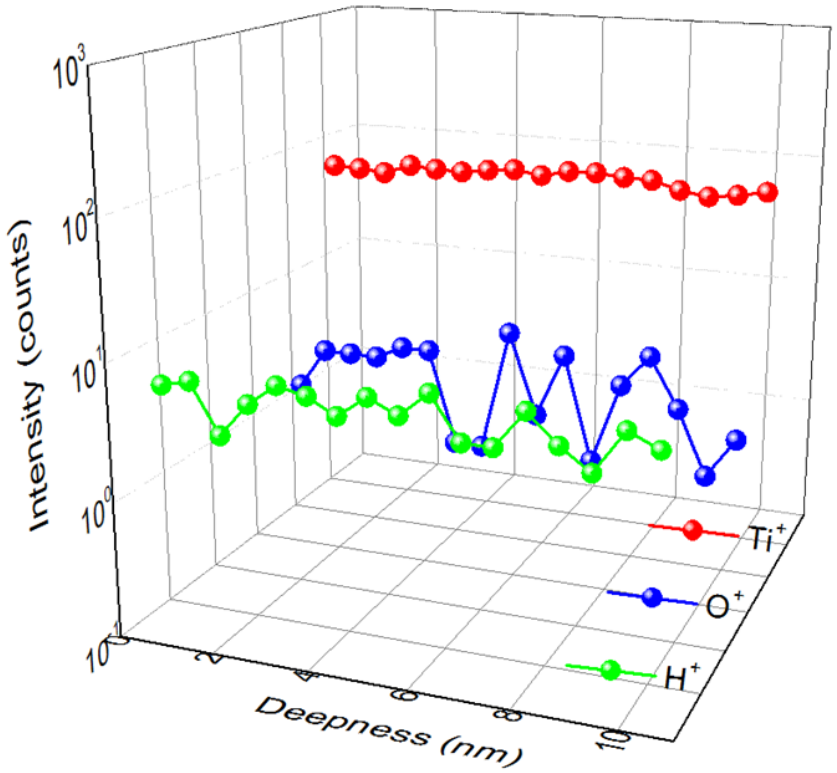

Supplementary Figure 7 | TOF-SIMS profile of a 10 nm thick hydrated nanocomposite (*HN*) nanosheet.

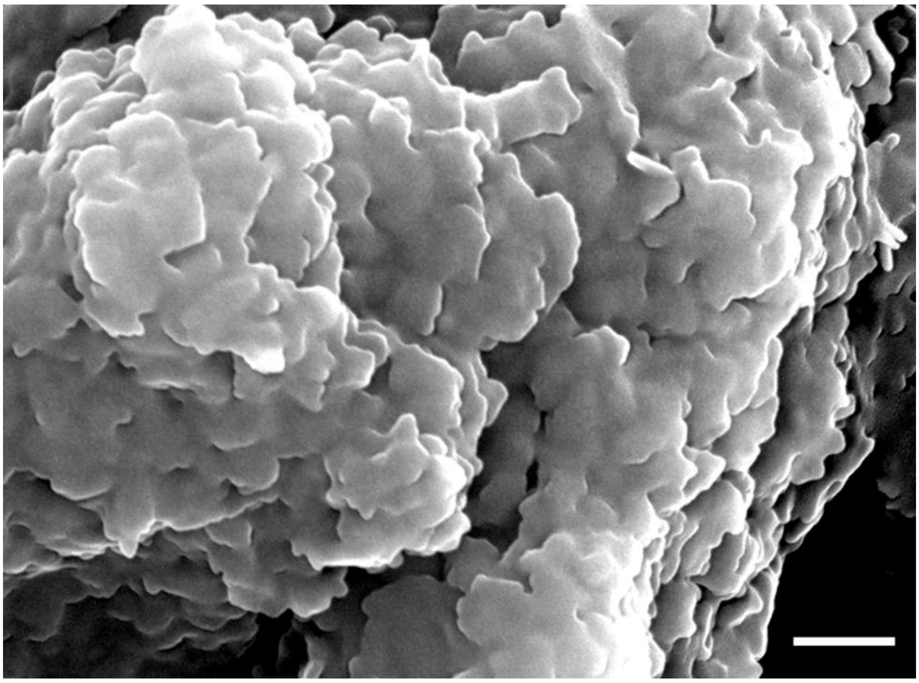

Supplementary Figure 8 | SEM image of layered structure (*LS*). *Scale bar*, 200 nm.

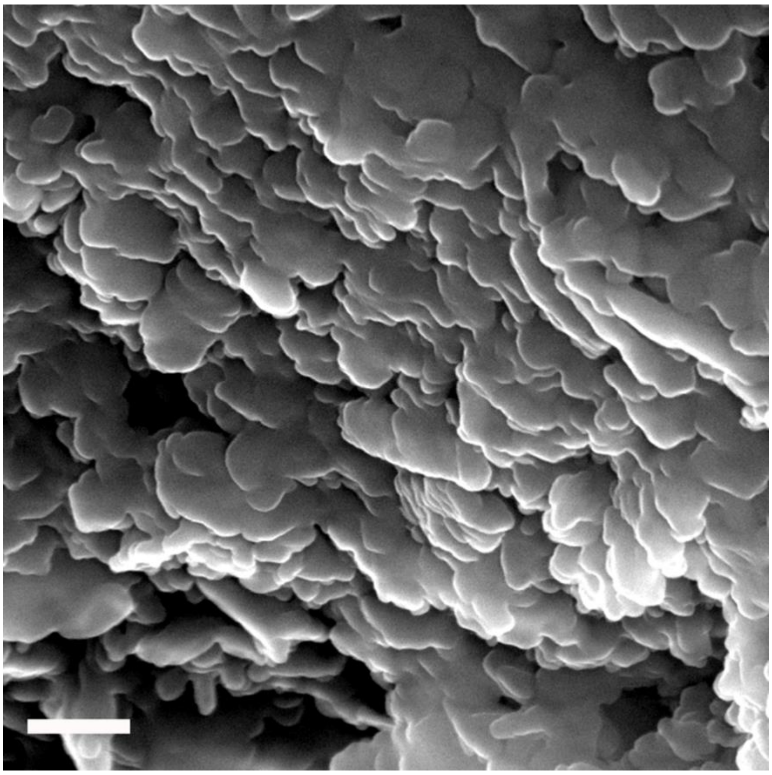

Supplementary Figure 9 | SEM image of hydrated nanocomposite (*HN*). *Scale bar*, 200 nm.

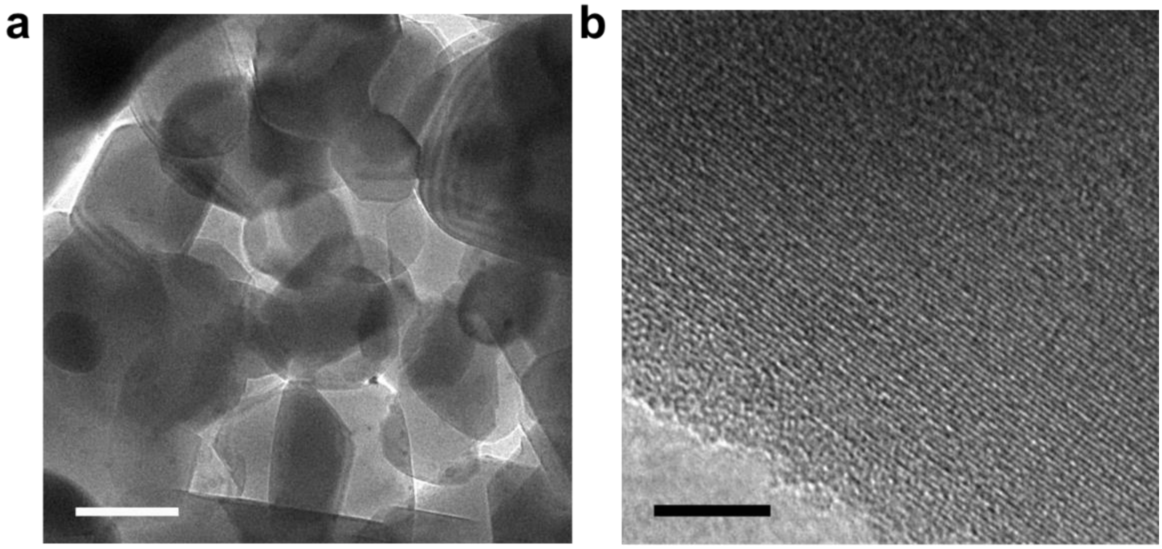

Supplementary Figure 10 | HRTEM images of dry nanocomposite (*DN*). *Scale bar*, 100 nm (a), 5 nm (b).

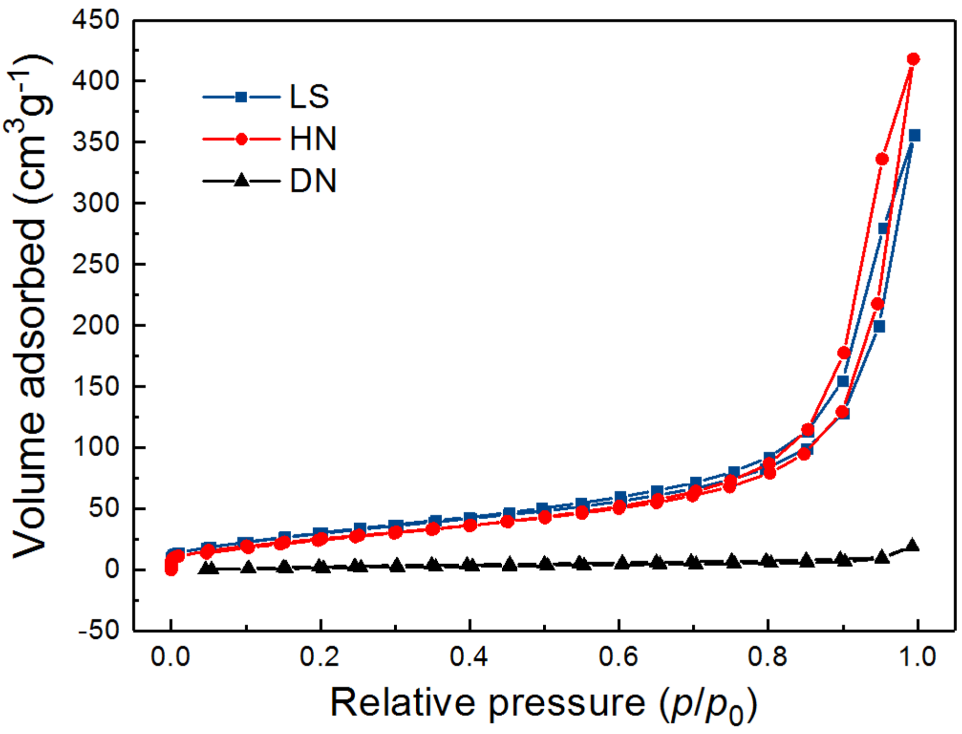

Supplementary Figure 11 | N_2 adsorption/desorption isotherms of layered structure (*LS*), hydrated nanocomposite (*HN*) and dry nanocomposite (*DN*).

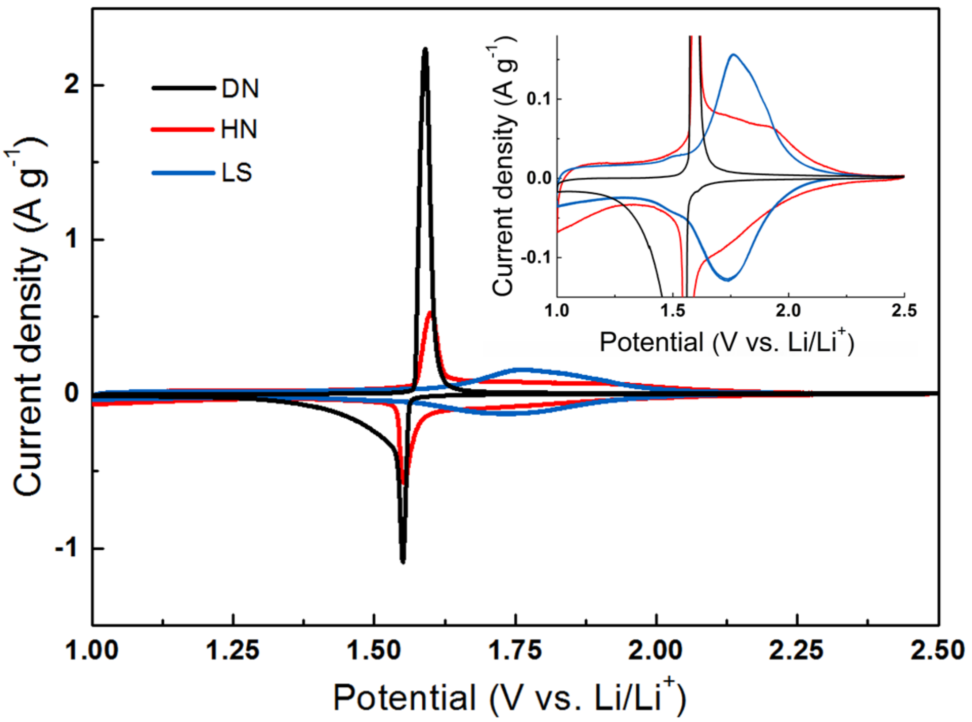

Supplementary Figure 12 | Cyclic voltammetry curves at 0.1 mV s^{-1} for comparison among layered structure (*LS*), hydrated nanocomposite (*HN*) and dry nanocomposite (*DN*) electrode materials.

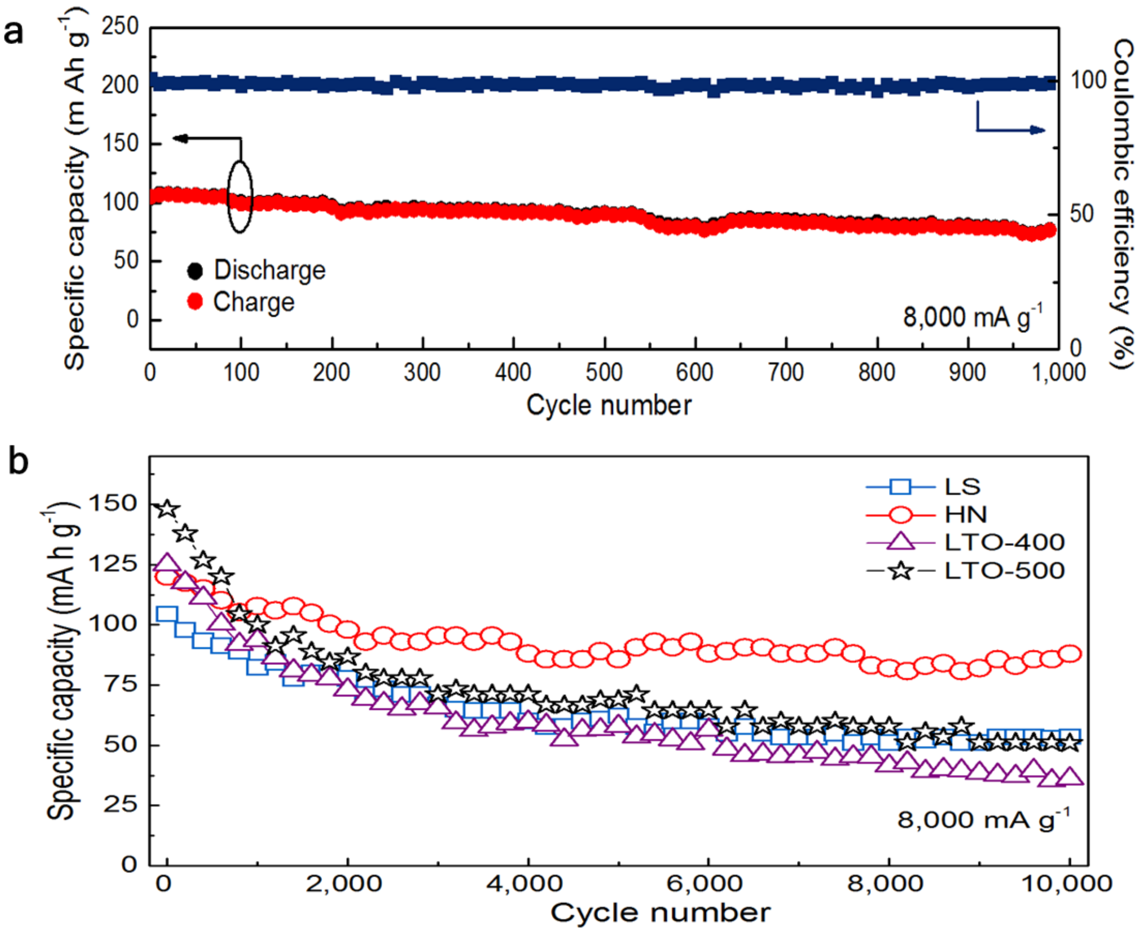

Supplementary Figure 13 | Electrochemical performances of as-synthesized electrode materials. (a) Cycling stability and Coulombic efficiency of layered structure (*LS*) electrodes and (b) the comparison of cycling performances at current density of 8,000 mA g^{-1}.

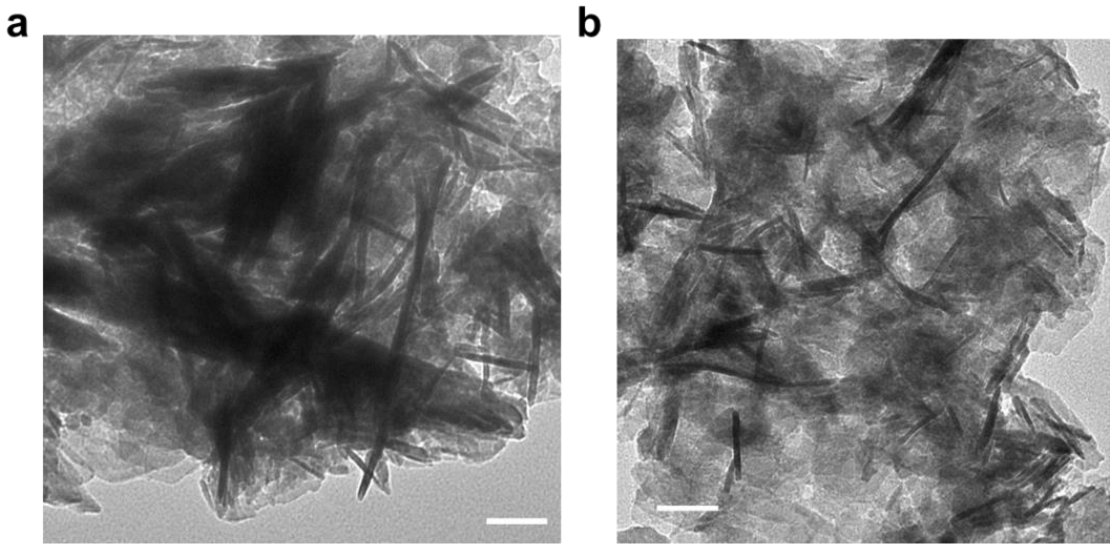

Supplementary Figure 14 | TEM images of hydrated nanocomposite (*HN*) after 10,000 cycles. *Scale bar*, 50 nm (a, b).

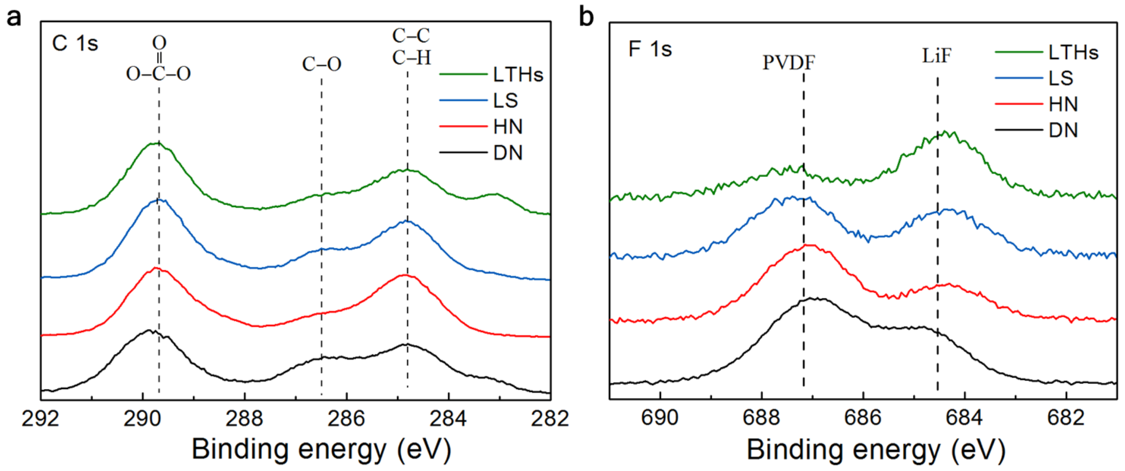

Supplementary Figure 15 | XPS spectra analysis of as-synthesized electrodes. C 1*s* (a) and F 1*s* (b) XPS spectra of lithium titanate hydrates (*LTHs*), layered structure (*LS*), hydrated nanocomposite (*HN*) and dry nanocomposite (*DN*) electrodes after 10,000 cycles at 4,000 mA g^{-1}.

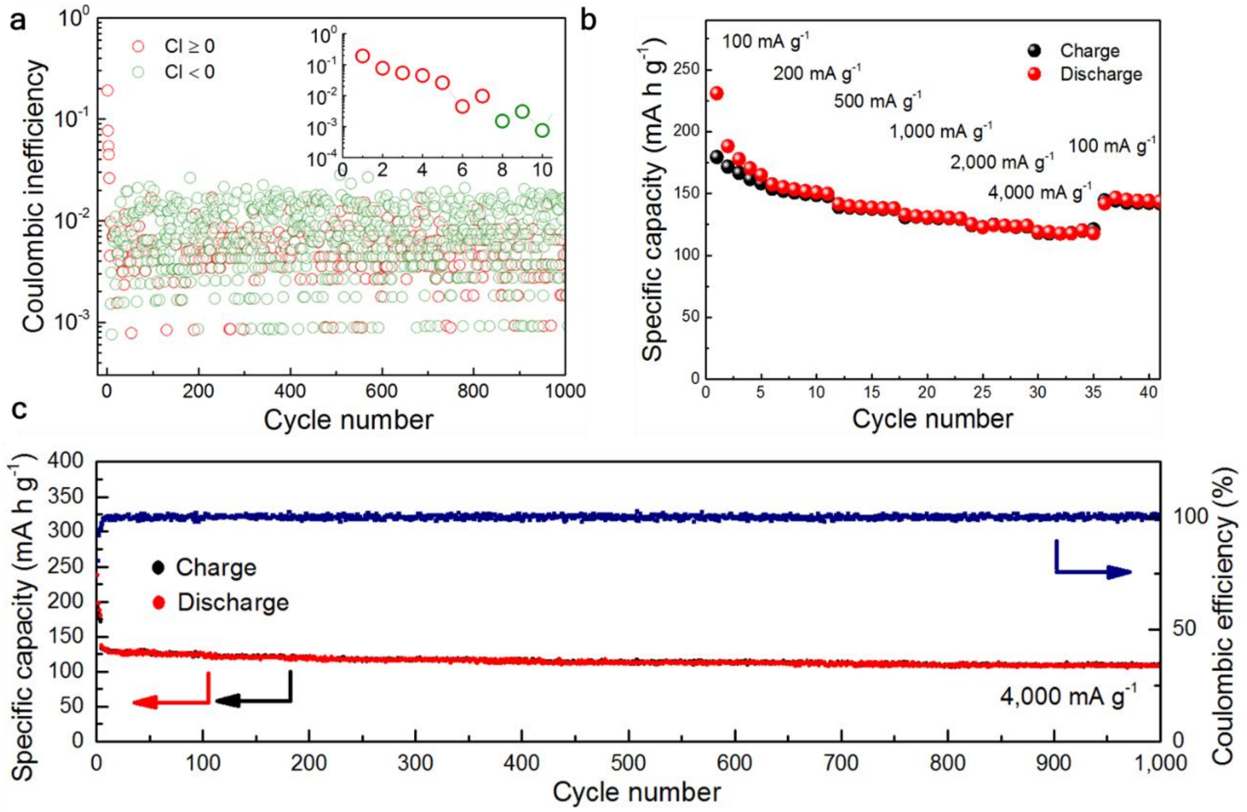

Supplementary Figure 16 | Electrochemical performances of hydrated nanocomposite (*HN*) when 1M LiTFSI in EC and DMC (1:1 by volume) was chosen as the electrolyte. (a) Coulombic inefficiency at 4,000 mA g^{-1}, the insert magnifies Coulombic inefficiency for the first 10 cycles; (b) The rate performances and (c) Cycle stability and Coulombic efficiency at 4,000 mA g^{-1}.

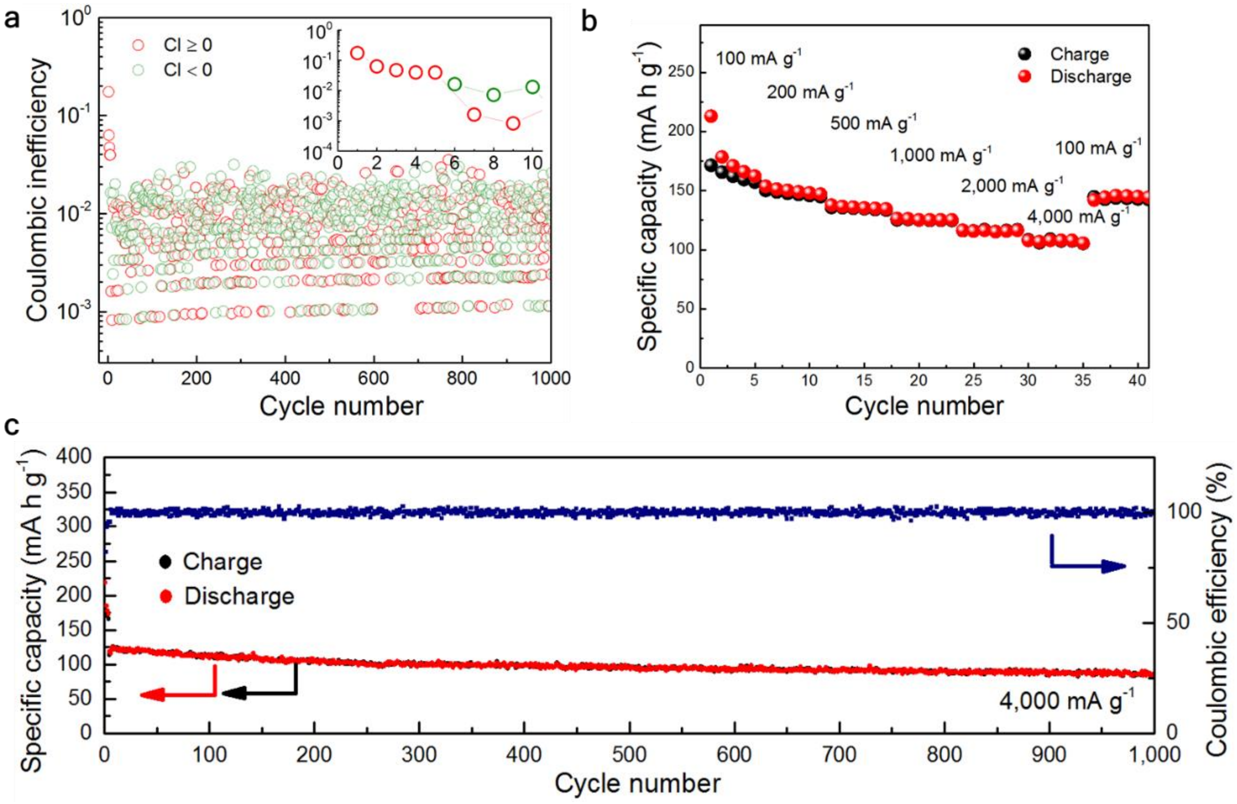

Supplementary Figure 17 | Electrochemical performances of layered structure (*LS*) when 1M LiTFSI in EC and DMC (1:1 by volume) was chosen as the electrolyte. (a) Coulombic inefficiency at 4,000 mA g^{-1}, the insert magnifies Coulombic inefficiency for the first 10 cycles; (b) The rate performances and (c) Cycle stability and Coulombic efficiency at 4,000 mA g^{-1}.

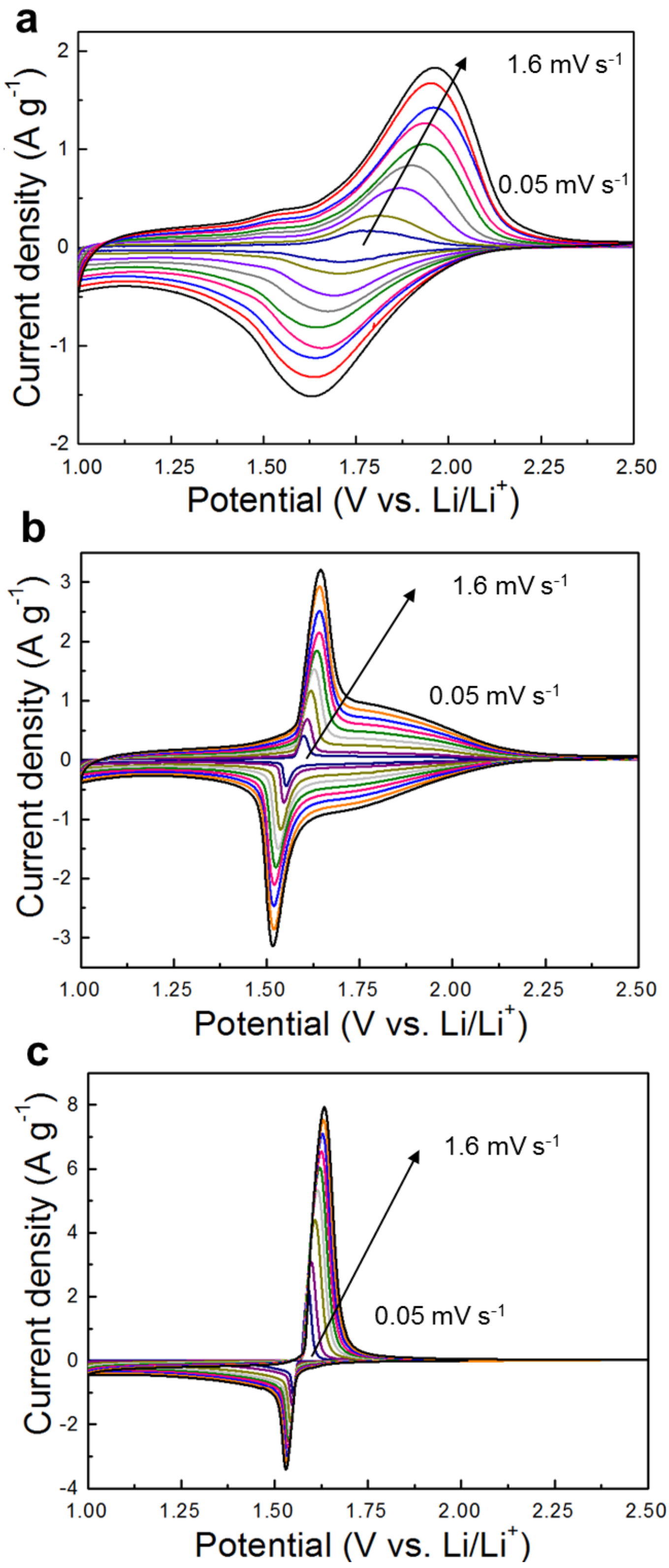

Supplementary Figure 18 | Cyclic voltammetry curves of as-synthesized electrodes from 0.05 to 1.6 mV s^{-1}. (a) Layered structure (*LS*), (b) hydrated nanocomposite (*HN*) and (c) dry nanocomposite (*DN*) electrodes.

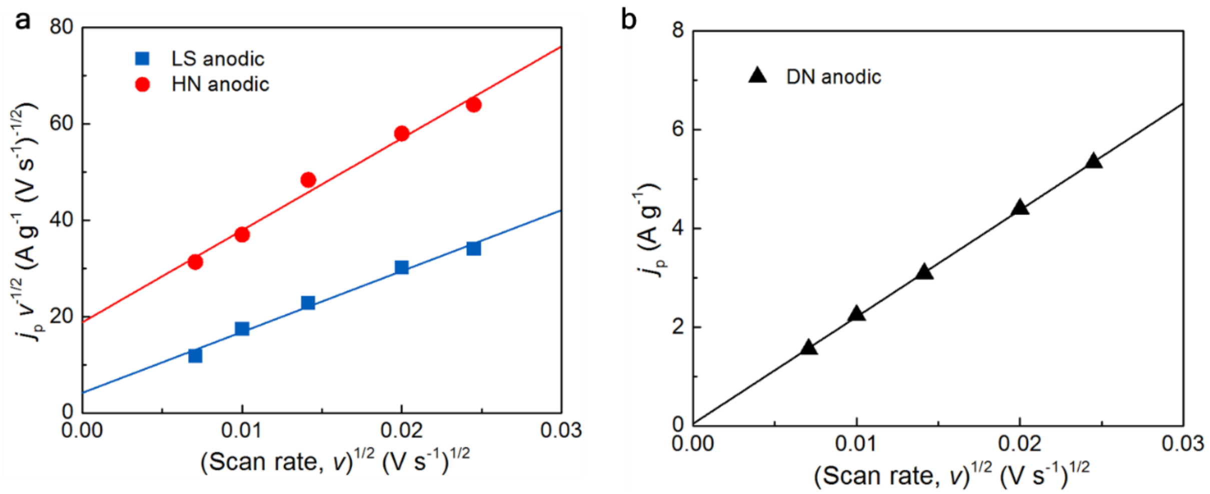

Supplementary Figure 19 | Linear fitting to j_p $v^{-1/2}$ *vs.* $v^{1/2}$ for layered structure (*LS*) and hydrated nanocomposite (*HN*) electrode materials (a) and linear fitting to j_p *vs.* $v^{1/2}$ for dry nanocomposite (*DN*) electrode materials (b).

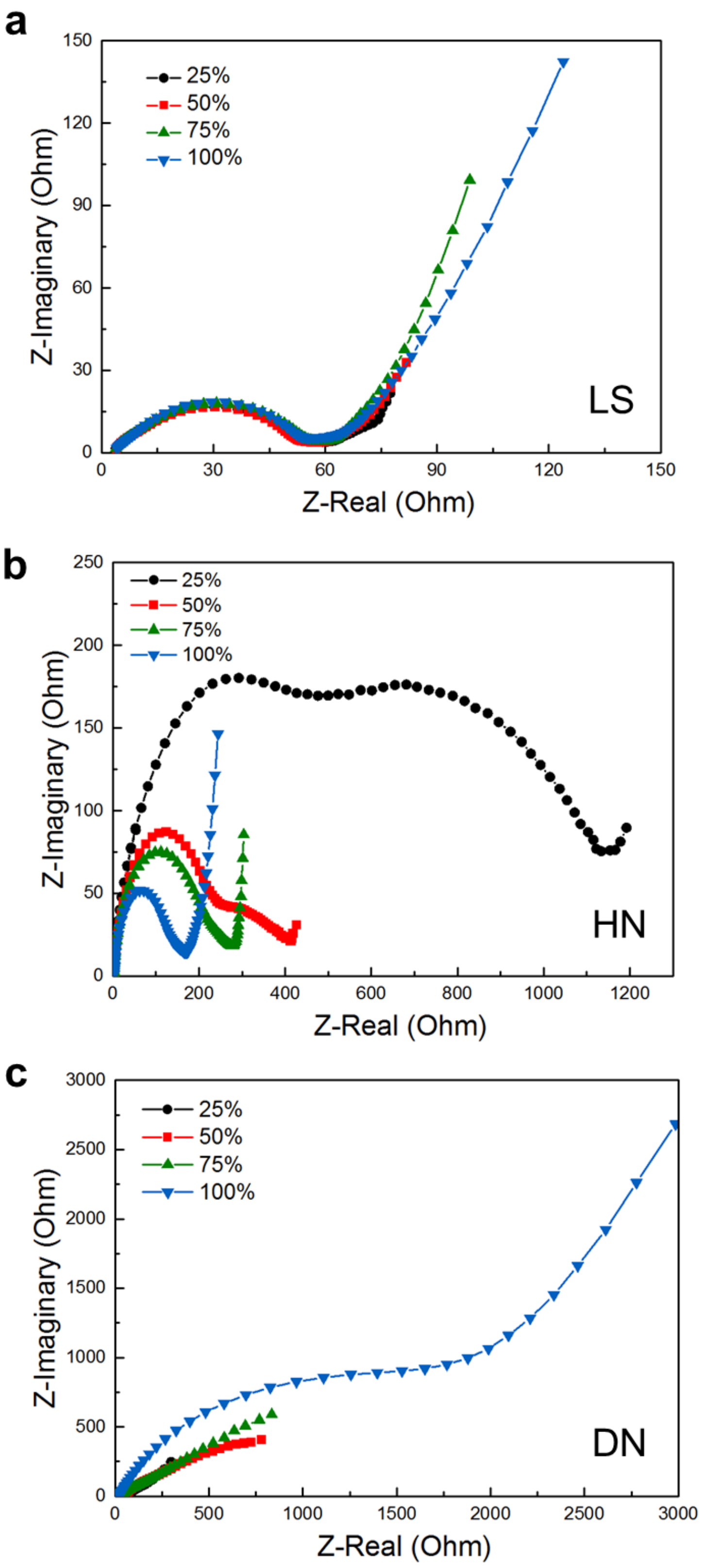

Supplementary Figure 20 | EIS analysis of as-synthesized electrodes measured at different depths of discharge. (a) Layered structure (*LS*), (b) hydrated nanocomposite (*HN*) and (c) dry nanocomposite (*DN*) electrodes.

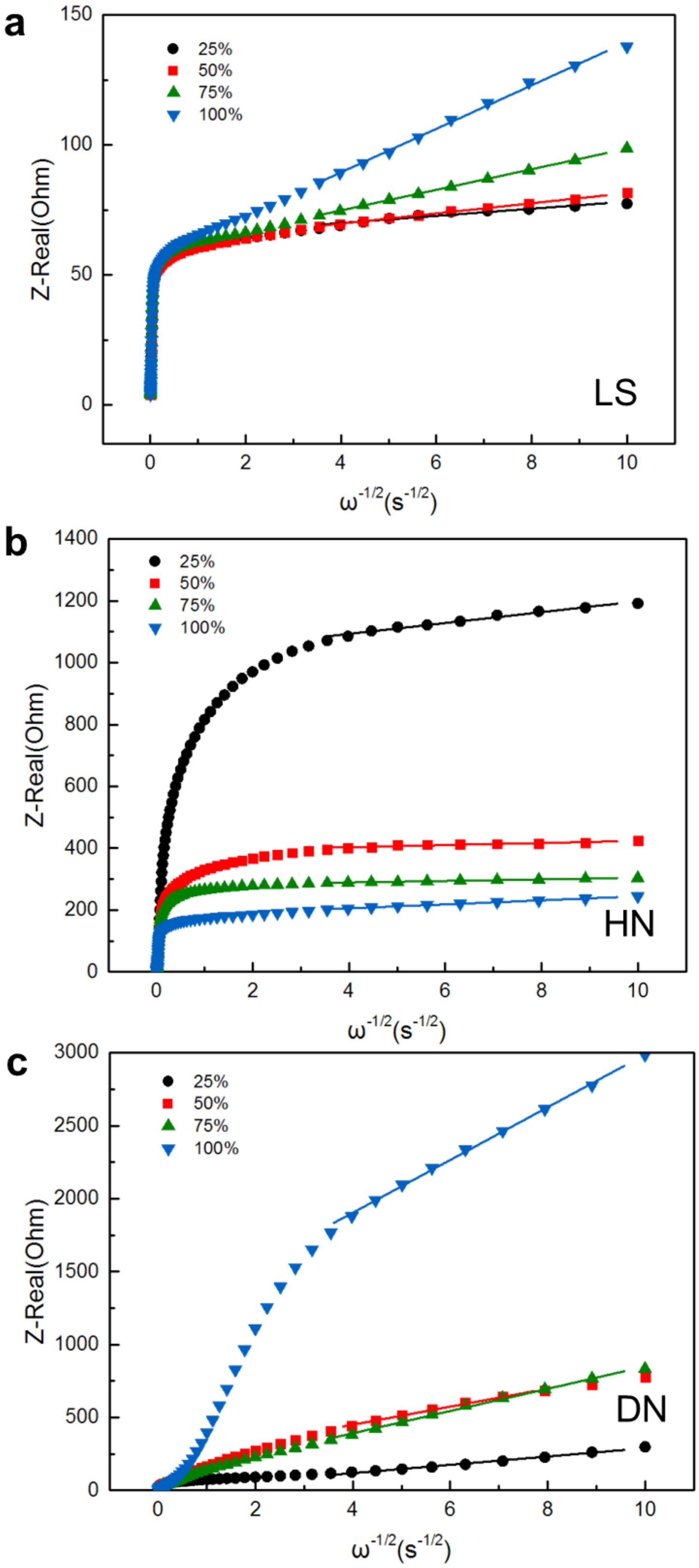

Supplementary Figure 21 | Z' *vs.* $\omega^{-1/2}$ plots of as-synthesized electrodes in the low frequency region obtained from EIS measurements. (a) Layered structure (*LS*), (b) hydrated nanocomposite (*HN*) and (c) dry nanocomposite (*DN*) electrodes.

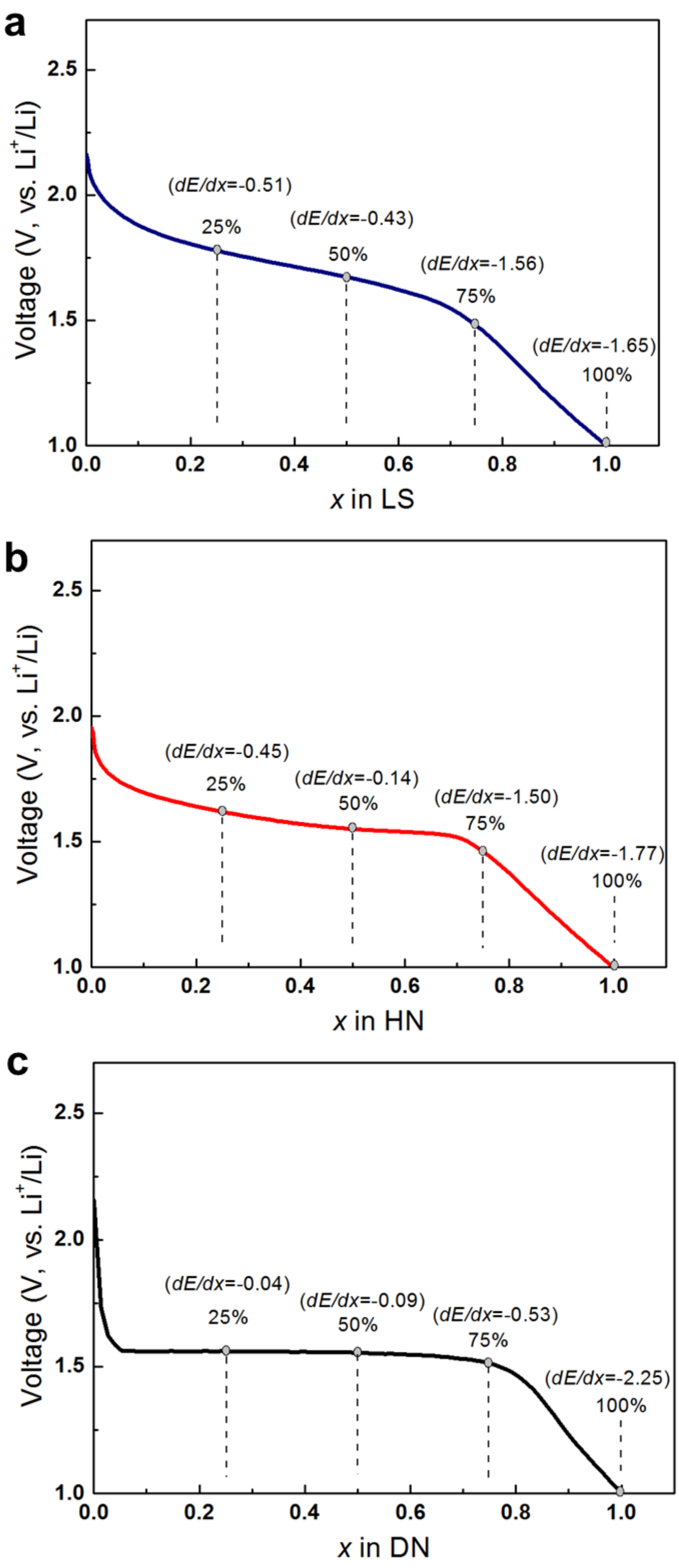

Supplementary Figure 22 | Discharge curves of as-synthesized electrodes at a current density of 50 mA g^{-1}. (a) Layered structure (*LS*), (b) hydrated nanocomposite (*HN*) and (c) dry nanocomposite (*DN*) electrodes.

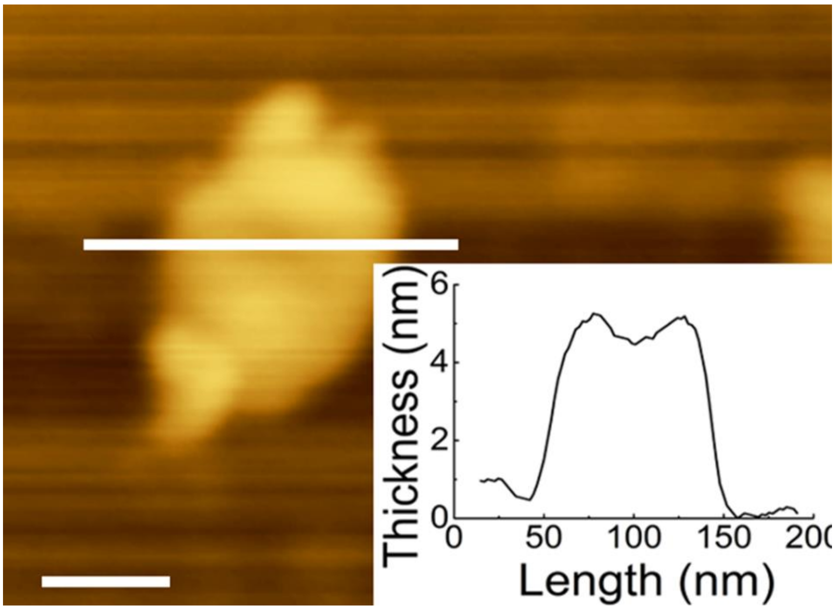

Supplementary Figure 23 | AFM image of hydrated nanocomposite (*HN*). The insert shows its corresponding thickness analysis conducted along the white line. *Scale bar*, 100 nm.

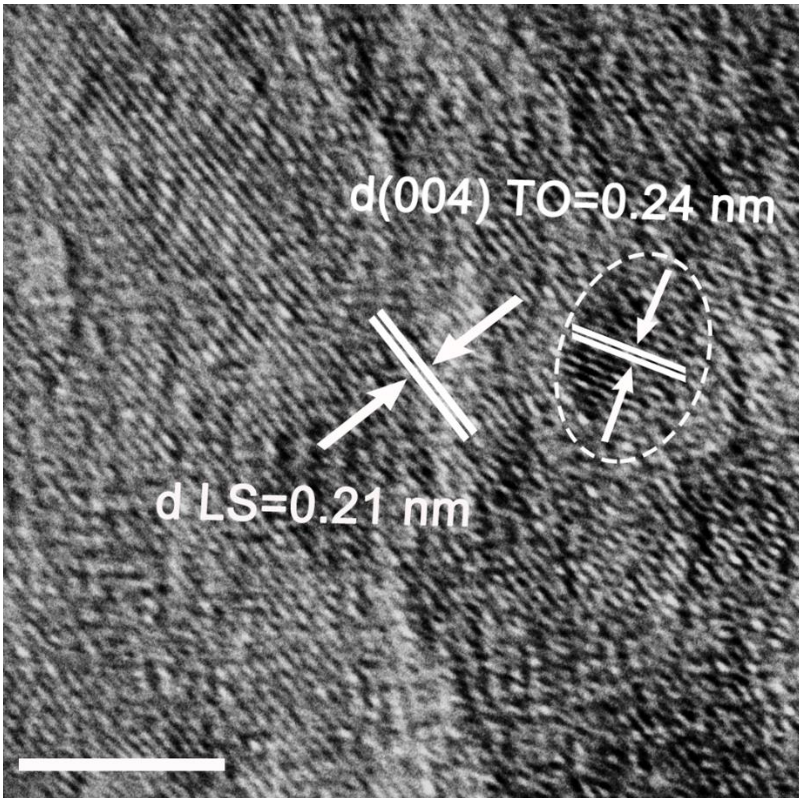

Supplementary Figure 24 | HRTEM image of a hydrated nanocomposite (*HN*) with a spacing of 0.21 nm for layered structure (*LS*) and 0.24 nm for anatase TiO_2. *Scale bar*, 100 nm.

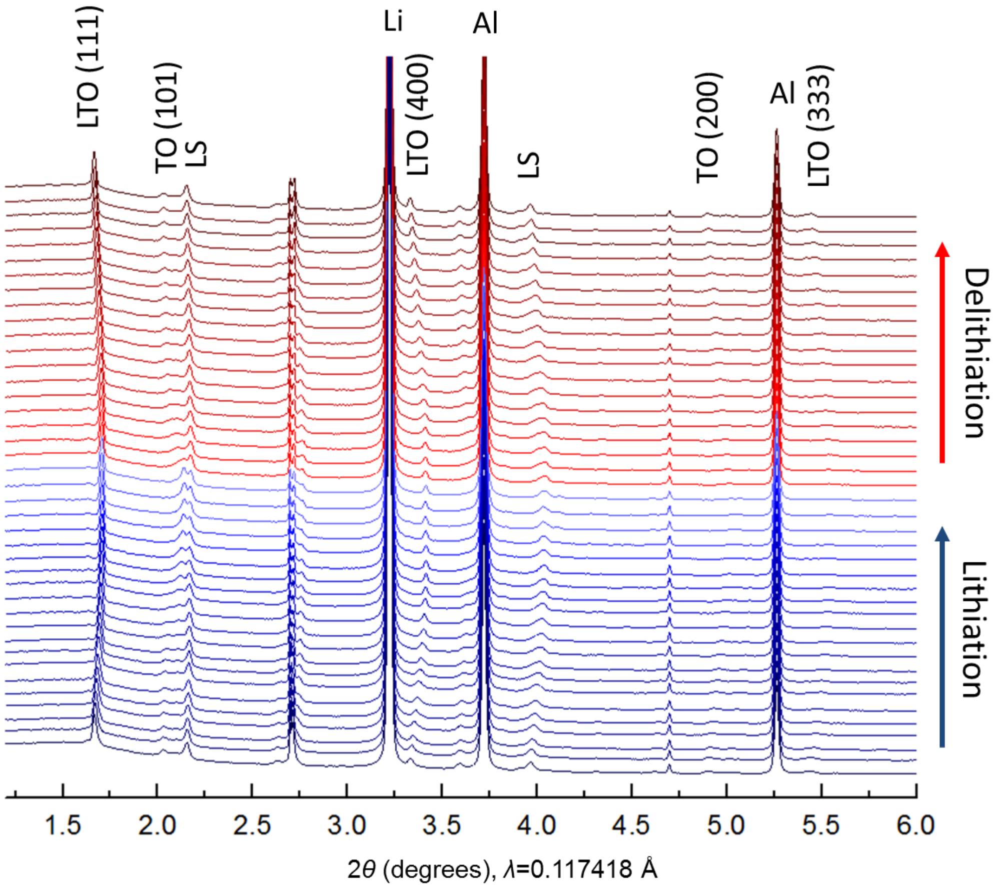

Supplementary Figure 25 | *In situ* synchrotron XRD results during the third cycle of hydrated nanocomposite (*HN*) electrode cycled at 100 mA g^{-1}.

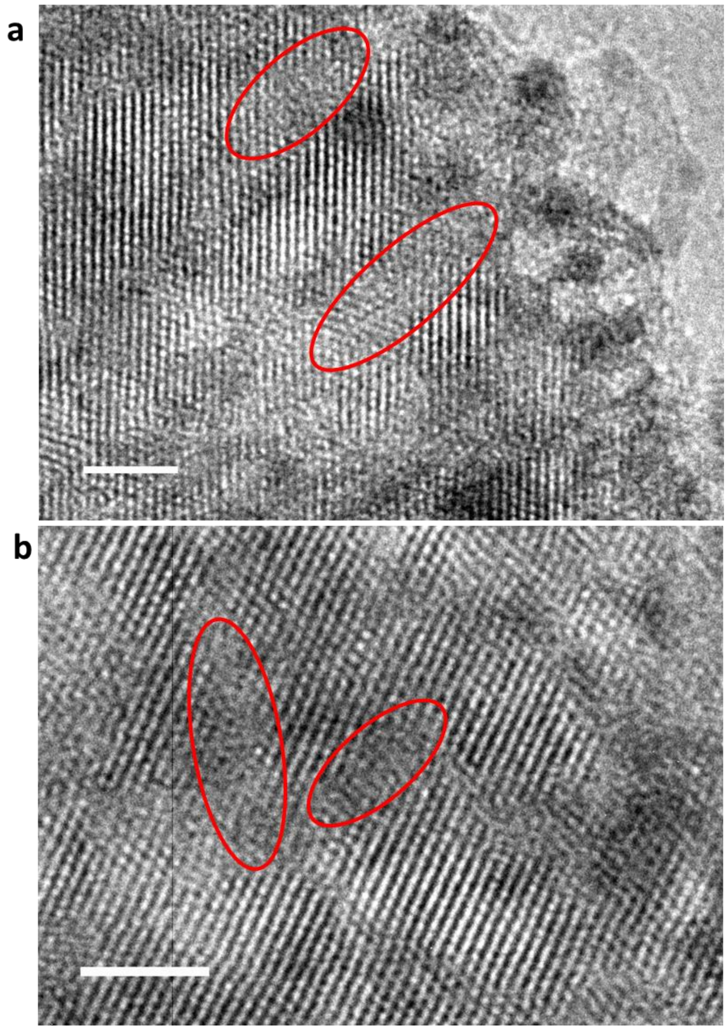

Supplementary Figure 26 | HRTEM images of hydrated nanocomposite (*HN*). The lattice distortion and disorder at the interfaces are illustrated in red circles. *Scale bar*, 5 nm (a, b).

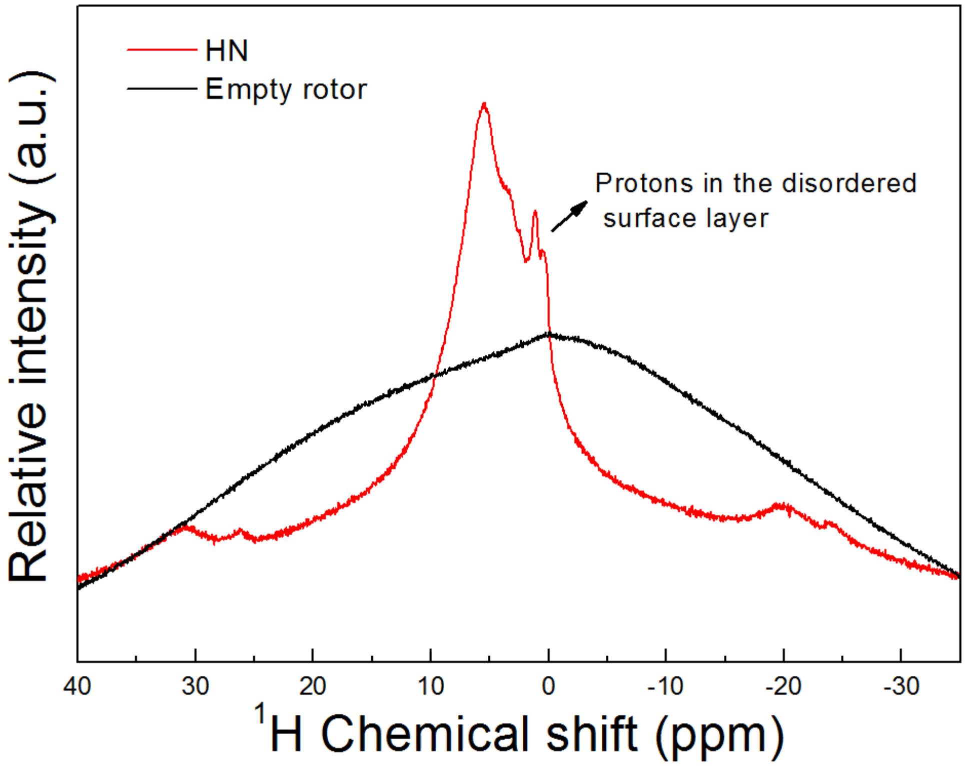

Supplementary Figure 27 | ^{1}H solid state MAS-NMR spectra of hydrated nanocomposite (*HN*).

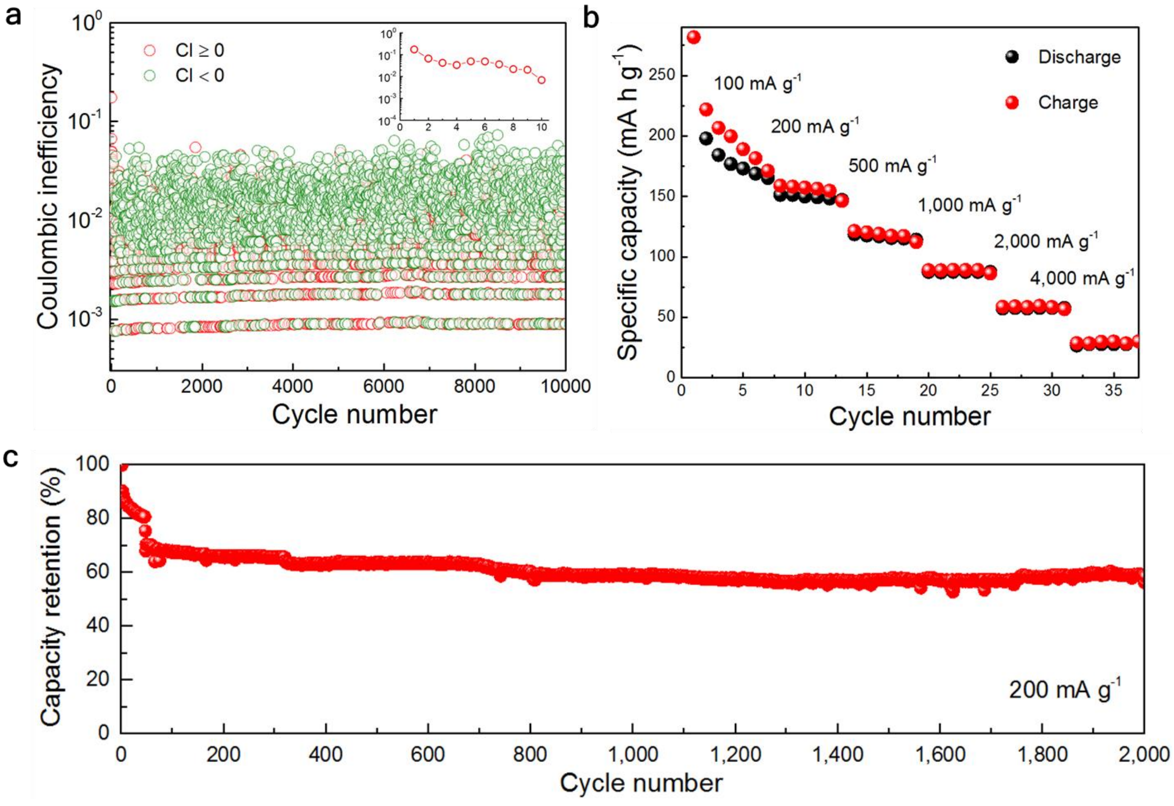

Supplementary Figure 28 | Electrochemical performances of hydrated nanocomposite (*HN*). (a) Coulombic inefficiency of hydrated nanocomposite (*HN*) at 4,000 mA g^{-1}, the insert magnifies CI for the first 10 cycles. (b) The rate performances and (c) capacity retention at 200 mA g^{-1} for $LiFePO_4$ *vs.* HN full batteries.

Supplementary Table 1 | Comparison of the electrochemical performances of the as-prepared lithium titanate hydrates with previously reported high-rate anode materials on the Li_2O-TiO_2 axis. Their electrode compositions are listed using the mass ratio of active materials: conductive carbon: binder.

Materials	Synthesis temperature (°C)	Electrode compositions	Loading density (mg cm^{-2})	High rate capacity (mAh g^{-1})	Cycling capacity retention after cycles	References
Lithium titanate hydrates (HN)	260	80:10:10	~1.0	124 (~70C)	86%, 10,000 cycles (~35C)	this work
Lithium titanate hydrates (LS)	190	80:10:10	~1.0	91 (~70C)	76%, 1,000 cycles (~70C)	this work
$Li_4Ti_5O_{12}$ nanowires without carbon coating	500~700	100:0:0	N/A	119 (30C)	80%, 100 cycles (5C)	1
$Li_4Ti_5O_{12}$ nanosheet arrays without carbon coating	550	100:0:0	0.04	78 (200C)	92.5%, 3,000 cycles (50C)	2
Large-pore Mesoporous $Li_4Ti_5O_{12}$ thin film without carbon coating	650	100:0:0	0.05	155 (32C)	95%, 3,000 cycles (64C)	3
$Li_4Ti_5O_{12}$ nanosheets with N-doped carbon coating	600	100:0:0	N/A	131 (100C)	99%, 100 cycles (10C)	4
Self-supported $Li_4Ti_5O_{12}$-C nanotube arrays	550	100:0:0	0.42	80 (100C)	93%, 500 cycles (10C)	5
Porous $Li_4Ti_5O_{12}$ without carbon coating	700~800	85:10:5	0.8~1.0	105 (30C)	98%, 500 cycles (5C)	6
Mesoporous $Li_4Ti_5O_{12}$ hollow spheres without carbon coating	500	70:20:10	N/A	104 (20C)	88%, 300 cycles (5C)	7
Mesoporous $Li_4Ti_5O_{12}$ with carbon coating	700	80:10:10<br>90:0:10	N/A	115 (10C)<br>90 (5C)	90%, 500 cycles (10C)	8
Porous $Li_4Ti_5O_{12}$ spheres with N-doped carbon coating	600	80:10:10	N/A	130 (10C)	83%, 2200 cycles (2C)	9
Compact $Li_4Ti_5O_{12}$ spheres with carbon coating	700	80:10:10	1.5	148.6 (10C)	97.3%, 500 cycles (10C)	10
Ti^{3+}-free three-phase $Li_4Ti_5O_{12}/TiO_2$	500	80:10:10	1.0	156 (50C)	80%, 1000 cycles (25C)	11
$Li_4Ti_5O_{12}$ with mesoporous carbon matrix	750	90:2:8	N/A	92.6 (40C)	94.4%, 1,000 cycles (20C)	12
$Li_4Ti_5O_{12}$ micro-spheres/ carbon textiles	550	80:12:8	N/A	119 (60C)	94.7%, 200 cycles (10C)	13
Tailored $Li_4Ti_5O_{12}$/ cathodically induced graphene composite	800	80:10:10	1.3	126 (100C)	74.6%, 500 cycles (25C)	14
Monodisperse $Li_4Ti_5O_{12}$ nanospheres	750	80:10:10	~2.0	108.9 (80C)	92.6%, 500 cycles (10C)	15
Templated spinel $Li_4Ti_5O_{12}$	--	90:5:5	0.8	85 (200C)	--	16
Elongated bending TiO_2(B) nanotube without carbon coating	400	100:0:0	N/A	164 (15C)	~70%, 10,000 cycles (25C)	17
Mesoporous TiO_2(B) microspheres without carbon coating	500	75:15:15	1~2	115 (60C)	90%, 5,000 cycles (10C)	18
Porous TiO_2(B) nanosheets without carbon coating	350	70:20:10	~1	202 (10C)	92.6%, 200 cycles (10C)	19
TiO_2(B) nanosheets on active carbon fabric	350	80:10:10	1.0~1.2	97 (30C)	87%, 2,000 cycles (20C)	20
Gel-like TiO_2-based nanotubes	500	100:0:0	0.3~0.5	133 (30C)	86%, 6,000 cycles (30C)	21
Ti^{3+} self-doped rutile TiO_2 nanorods	N/A	75:15:15	1.0~1.2	93.6 (50C)	98.4%, 1,000 cycles (50C)	22

Supplementary Table 2 | The intensity value for TOF-SIMS profile in Supplementary Figure 7.

Deepness (nm)	H	O	Ti
1.18	8.00	2.00	81.11
1.77	9.00	4.00	79.11
2.36	4.00	4.00	76.10
2.95	7.00	4.00	90.13
3.54	10.00	5.00	87.12
4.13	9.00	5.00	86.12
4.72	7.00	1.00	92.15
5.31	10.00	1.00	96.15
5.9	8.00	8.00	90.14
6.49	12.00	2.00	100.18
7.08	6.00	6.00	101.17
7.67	6.00	1.00	97.17
8.26	11.00	4.00	96.15
8.85	7.00	7.00	84.12
9.44	5.00	3.00	78.10
10.03	10.00	1.00	83.13
10.62	8.00	2.00	91.14

Supplementary Table 3 | The calculated relative ratio of pseudocapacitive and bulk diffusion currents (I_c: I_d) for layered structure (*LS*) and hydrated nanocomposite (*HN*) electrode materials at different sweep rates.

Sweep rate (mV s^{-1})	LS anodic	HN anodic
0.05	2.15	0.72
0.1	3.03	1.02
0.2	4.29	1.44
0.4	6.07	2.03
0.6	7.43	2.49

Supplementary Table 4 | The Li-ion diffusion coefficients of layered structure (*LS*), hydrated nanocomposite (*HN*) and dry nanocomposite (*DN*) electrodes at different depths of discharge marked in Supplementary Fig. 22.

Depth of discharge \ Diffusion coefficient	LS $cm^2\ s^{-1}$	HN $cm^2\ s^{-1}$	DN $cm^2\ s^{-1}$
**25%**	2.10×10^{-8}	2.72×10^{-10}	4.87×10^{-13}
**50%**	8.34×10^{-9}	2.93×10^{-10}	2.66×10^{-13}
**75%**	2.46×10^{-8}	1.00×10^{-9}	6.81×10^{-14}
**100%**	8.21×10^{-9}	1.17×10^{-8}	2.46×10^{-11}

Supplementary Note 1 (Supplementary Figure 4): A main peak at about 3400 cm^{-1} is associated with O–H stretching vibration and most probably as Ti–OH surface group.[23]

Supplementary Note 2 (Supplementary Figure 5): Even at relative low current density of 100 mA g^{-1}, the discharge specific capacity of LTHs-precursor electrodes plummeted from 258.5 mA h g^{-1} to 59.7 mA h g^{-1} for the first 50 cycles, showing an unsatisfactory cycling performance in aprotic electrochemical system.

Supplementary Note 3 (Supplementary Figure 7): It was measured on (100)-oriented silicon wafer showing the distribution of proton together with other elements, as a function of depth from the top surface. H, O and Ti elements exhibited some fluctuation from Supplementary Table 2, implying the step function in the layered structures.

Supplementary Note 4 (Supplementary Figure 11): Before the growth of $Li_4Ti_5O_{12}$-TiO_2 crystallites, LS has a relatively higher surface area of 120 m^2 g^{-1}. With the rising dehydration temperature, the surface area of HN decreased to 103 m^2 g^{-1}. At the end of the dehydration and phase transition progress, DN showed the lowest surface area of 10 m^2 g^{-1} caused by an ever-increasing aggregation or coarsening of nanoparticles.

Supplementary Note 5 (Supplementary Figure 12): The dominated pairs of sharp redox peaks at about 1.73 V and 1.44 V corresponding to the diffusion-controlled process in $Li_4Ti_5O_{12}$ phase, while the pseudocapacitive behavior of LS at around 1.75 V and the diffusion-controlled behavior of TiO_2 at 1.7 V/2.0 V were superimposed upon each other to produce two flat and wide peaks.

Supplementary Note 6 (Supplementary Figure 13): The lithium titanate anode LTO-400 and LTO-500 were also synthesized by heating the LTHs precursor at 400 °C and 500 °C, respectively, and kept the other experimental variables fixed. The electrochemical performances among LS, HN, LTO-400 and LTO-500 materials was illustrated in Supplementary Fig. 13b. LTO-400 can deliver more specific capacity (125 mAh g^{-1}) in the initial cycle compared to LS and HN, however, its capacity drops from 118 mAh g^{-1} to 66 mAh g^{-1} in the first three thousand cycles and then tapers

off to 36 mAh g^{-1} in the following several thousand cycles. Similarly, the capacity of LTO-500 plummets from 148 mAh g^{-1} to 74 mAh g^{-1} in the first three thousand cycles and maintains only 51 mAh g^{-1} after 10,000 cycles. The reason is that $Li_4Ti_5O_{12}$ and TiO_2 with mainly diffusion-controlled redox reaction can usually storage more Li-ions than LS with mainly surface-controlled redox reaction, but the Li-ion diffusion coefficient of $Li_4Ti_5O_{12}$ and TiO_2 is lower than the 2D materials of LS and HN, resulting in the relatively unsatisfactory cycling performances of LTO-400 and LTO-500 electrodes.

Supplementary Note 7 (Supplementary Figure 15): The XPS was conducted to analyze the surface status and composition of LHTS, LS, HN and DN electrodes after 10,000 cycles at 4000 mA g^{-1}. The electrodes were carefully separated from the other components of the coin cell and washed with DMC to remove the electrolyte. All the operations were conducted in a glove box under Argon atmosphere.

In the comparison of C 1*s* spectrum (Supplementary Fig. 15a), three main peaks at 289.8, 286.5 and 284.8 eV can be assigned to carbon atoms in a three-oxygen environment (CO_3-like), one-oxygen environment (CO-like), carbon atom bound only to C or H atoms, respectively. These three carbon species could demonstrate the presence of Li_2CO_3 and lithium alkyl carbonate (ROCOOLi) well.[24,25] The possible SEI formation mechanisms are as follows: i) intrinsic catalysis by Ti^{4+} species during the discharge process, Ti^{3+} is oxidized to Ti^{4+}, whereby the latter species may have a catalytic influence on the electrolyte decomposition (Ti^{4+} + EC/PC/DMC→Ti^{3+} + radicals + CO_2); ii) electrolyte (EC) reduction from lithiated LTO ($2Li^+$ + EC→Li_2CO_3 / $(CH_2CH_2OLi)_2$ + C_2H_4).[26, 27]

In the comparison of F 1*s* spectrum (Supplementary Fig. 15b), two peaks at 687.1 and 684.6 eV can be assigned to PVDF and LiF, respectively. For DN which is completely water-free, a small amount of the LiF was observed which could be caused by some possibilities as follows: a) the hydrolysis of $LiPF_6$ ($LiPF_6$+H_2O→LiF+POF_3+2HF), as water unavoidably exists in a very low concentration (ppm) in the electrolyte; b) $LiPF_6$ salt would decompose by itself during reduction in the charge/discharge cycles ($LiPF_6$→LiF+PF_5); c) salt reaction with products such as Li_2CO_3, resulting from reaction (PF_5+Li_2CO_3→2LiF+POF_3+CO_2, $LiPF_6$+Li_2CO_3→3LiF +POF_3+CO_2, et al).[28,29] The intensity of LiF peak gradually increased from HN to LS, which implied that more

structural water might be broken and released into the electrolyte during long-term and super-fast cycling process, resulting in slight hydrolysis of $LiPF_6$. This could be the reason of the slight capacity fading of LS and HN. As a contrast, there shows much more LiF at the surface of LTHs electrodes due to the strong peak of LiF. This result demonstrate that the electrodes with more loosely bound water (such as crystallographic water) would be more likely to cause the decomposition of $LiPF_6$, leading to electrochemical performances worsened rapidly in aprotic electrolyte.

Supplementary Note 8 (Supplementary Figure 16, 17): For quite some time, the researchers considered that PF_5, a strong Lewis acid and one of the decomposition products of $LiPF_6$, is the major source of the side reactions in the presence of trace amount of water. According to the analysis about formation mechanism of SEI film above, many recent reports have illustrated that the electrolyte (say, EC/PC/DMC) decomposition by Ti^{4+} species and electrolyte (say, EC) reduction from lithiated LTO are the main side reactions for Ti-based materials during charging/discharging process.[26,27] We have designed the following experiments using LiTFSI as electrolyte salts (1M LiTFSI in EC and DMC (1:1 by volume)) to support the opinion mentioned above. As LiTFSI salt is not sensitive to water, therefore if the side reactions will still happen between the electrode and electrolyte, there could be some else decomposition mechanism resulting in unsatisfactory "Coulombic inefficiency" (CI).[30]

We further tested some electrochemical performances of HN when using different lithium salts (LiTFSI and $LiPF_6$). The CI of HN electrode with LiTFSI electrolyte (hereinafter refer as HN-LiTFSI, Supplementary Fig. 16) is quite similar with that of HN electrode with $LiPF_6$ electrolyte (hereinafter refer as HN-$LiPF_6$), especially for the first 10 cycles, implying that the main decomposition mechanism may not be the hydrolysis of $LiPF_6$ with trace amount of water. Besides, the rate performance as well as cyclability of HN-LiTFSI are also similar with HN-$LiPF_6$. This phenomenon could demonstrate that the SEI formed after several cycles on the surface of HN with LiTFSI electrolyte is as stable as the one formed on the surface of HN with $LiPF_6$ electrolyte, thus both two HN electrodes with different lithium salts could exhibit excellent high-rate capacity and cycling stability. In the same way, we also illustrated electrochemical performances of LS when using LiTFSI as lithium salts (Supplementary Fig. 17). The result and trend are the same as

the condition of HN discussed above.

Supplementary Note 9 (Supplementary Figure 18): The contribution of pseudocapacitity and diffusion-controlled capacity was further revealed by analyzing the cyclic voltammetry data (in Supplementary Figure 18) and can be quantified according to the following Equation (1-2):[31,32]

$$j_p = k_1 v + k_2 v^{1/2} \qquad (1)$$

or

$$j_p\, v^{-1/2} = k_1 v^{1/2} + k_2 \qquad (2)$$

where $k_1 v$ represents pseudocapacitive current (I_c) contributed by interfacial storage and bulk faradic pseudocapacitance; $k_2 v^{1/2}$ represents the (bulk) diffusion current (I_d). The coefficients k_1 and k_2 can be determined by linear fitting to $j_p\, v^{-1/2}$ *vs.* $v^{1/2}$ in Supplementary Fig. 19a. Therefore, it is possible to calculate the relative contribution of I_c and I_d at specific scan rate (v).

Supplementary Note 10 (Supplementary Figure 19): The results of I_c: I_d for LS and HN electrodes are illustrated in Supplementary Table 3. It should be noted that I_c: I_d for LS is relatively high compared to that of HN, even at relatively low sweep rate of 0.05 mV s^{-1}. This result demonstrates that pseudocapacitive feature of LS, which is consistent with the electrochemical mechanism analysis in Figure 4a. For HN, at relatively low scan rate (0.05 mV s^{-1}), the relative ratio of I_c: I_d is 0.72, indicating the mixed pseudocapacitive and diffusion-controlled mechanism of HN. At the sweep rate of 0.1 mV s^{-1}, the pseudocapacitive storage current is approximately equal to the bulk storage current. The capacitive currents might be due to the synergistic effect of the pseudocapacitive feature of LS and nano-size effect of LTO and TO as well as large amount of interfaces among LTO-TO-LS. With a further increase of the sweep rate, the pseudocapacitive current eventually dominates the total current. For DN, the storage current density j_p illustrates a good linear relationship with $v^{1/2}$, implying that DN is totally diffusion controlled (Supplementary Fig. 19b).

Supplementary Note 11 (Supplementary Figure 20-22): The low-frequency Warburg contribution of the impedance response has been used to determine the Li-ion diffusion coefficient (D_{Li}) at various state of discharge in the electrodes (Supplementary Fig. 22). The expression for

D_{Li} can be written as:[33,34,35]

$$D_{\mathrm{Li}} = \frac{1}{2}\left[\left(\frac{V_{\mathrm{m}}}{FA\sigma_{\mathrm{w}}}\right)\frac{dE}{dx}\right]^{2} \qquad (3)$$

where V_m is the molar volume (we defined V_m for all three materials as 45.73 cm^3 mol^{-1} ($Li_4Ti_5O_{12}$) for simplification), F is the Faraday constant, A is the total contact area between the electrolyte and the electrode, and σ_{w} is the Warburg coefficient which was obtained from the Warburg region of impedance response. The σ_{w} values at different discharge depths (different potentials) can be obtained from the slope of lines in Z' *vs.* $\omega^{-1/2}$ plots (ω is the angular frequency) for the Warburg region. As shown in (Supplementary Fig. 21), the Z' *vs.* $\omega^{-1/2}$ plot for the low frequency Warburg region can be summarized as:

$$Z' = R + \sigma_{\mathrm{w}}\omega^{-1/2} \qquad (4)$$

The *dE/dx* obtained from discharge curve illustrates that the slopes of the discharge curve at 25%, 50%, 75% and 100% respectively (Supplementary Fig. 22). Each sample was activated for 10 cycles between 1.0-2.5V at 50 mA g^{-1} before test and stopped according to the percentage of the total discharge capacity (as shown in Supplementary Fig. 22). The EIS measurement was conducted right after the cell was stopped, and then it was charged/discharged for 2 complete cycles between 1.0-2.5V at 50 mA g^{-1} before the next measurement.

The reason why the Li-ion diffusion coefficients at 25%, 50% and 75% are lower than 100% is that 25%-75% of the HN and DN electrodes are associated the two-phase region, in which the total Li-ion insertion process is limited by Li-ion diffusion across the interface.[36] Therefore the Li-ion diffusion within the two-phase region is much lower than the Li-ion diffusion within the single-phase regions. However, there is a little change of D_{Li} for LS (8.21×10^{-9}-2.46×10^{-8} cm^2 s^{-1}), which might due to the surface-controlled feature of LS (here "surface" is taken to mean in the broad sense, that may include spacing between two adjacent LS monolayers).

Supplementary Note 12 (Supplementary Figure 25): Note that the LTO in our system reveal much larger lattice changes and we expected that this phenomenon might be ascribed to the size effect[37] and the existence of abundant lattice distortion and disorder[38] in HN composite.

Supplementary Note 13 (Supplementary Figure 27): From the result of blank rotor, no sharp

signals appear at low chemical shift. The large line width in the ^{1}H NMR spectra can be attributed to both dipole-dipole interactions and an intrinsic chemical shift spread, as proton bridging sites are located on different crystallographic planes in the structure. Two small sharpness ^{1}H NMR resonances at chemical shifts of 1.14 ppm and 0.56 ppm are associated with isolated OH groups without hydrogen bonds[39] and protons located in the disordered surface layer among $Li_4Ti_5O_{12}$-TiO_2-LS[40], respectively.

Supplementary Note 14 (Supplementary Figure 28): Herein, we will evaluate the possibility of in full batteries of HN material by using the concept of "Coulombic inefficiency".[30] In half cells, as the "live Li-ion" in pure lithium counter electrode is infinite compared to the working electrode, the electrochemical performance of working electrode is not affected by the CE or CI at all. However, when it comes to full cells, almost all the "live Li-ion" are from the cathode (i.e. $LiFePO_4$, $LiCoO_2$, *etc.*), if the CE (half cell) of the anode cannot be higher than 99.5% in less than 10 cycles, one solution is to use the slightly excess cathode as the supplement of "live Li-ion". However, if the $CE_{stabilized}$ is lower than 99.9% after 10 or more cycles, the "live Li-ion" would be consumed rapidly even if the cathode is several hundreds percent excess to the anode. In this situation, it is impossible to get a satisfactory cycling performance in full cells.

Supplementary Figure 28a illustrates calculated CI for HN electrode at 4,000 mA g^{-1}. The CI value for the first cycle is 10^{-1}, and then it dropped to $< 10^{-2}$ after 10th cycle, followed by fluctuating values distributed between 10^{-2} and 10^{-4} for the following 10,000 cycles. We note that there are more number of negative (green) CIs than positive (red) CIs, which means if a running-window average is done, the average CE actually exceeds 1 by a little. This may be explained by the reversible shuttling of soluble redox mediators in the electrolyte.[41] This result illustrated HN electrode could possess excellent SEI stability and highly reversible Li-ion insertion/extraction properties, because if the SEI were to fall off / regrow repeatedly with cycling, the average CE should be less than 1 (average CI should be positive). To further verify this, we assembled $LiFePO_4$ cathode *vs.* HN anode in full batteries using the same electrolyte in half cells and test their performances between 1.2 and 2.4 V. The full battery delivered specific capacity of 168, 149, 117, 89, 58 and 28 mA h g^{-1} in the 100, 200, 500, 1,000, 2,000 and 4,000 mA g^{-1} (Supplementary Fig. 28b). For the cycling performance at 200 mA g^{-1} in Supplementary Fig. 28c, 30% of the initial

capacity is lost in the first 50 cycles due to SEI growth and some side reactions for the electrodes. However, the cyclability tends to be quite stable for the next 2,000 cycles with only 10% of the initial capacity lost, indicating that HN is a very promising material as a LIB anode for the real-life energy storage application. It is true that the output voltage of the $LiFePO_4$ *vs.* HN full battery is somewhat low due to the relatively high potential (*vs.* Li) of HN compared with graphite anode. However, the advantages of improved safety over graphite as well as outstanding high-rate and long-cycling performances for lithium titanate hydrates are still obvious in some applications like electric buses, electric vans, smart grids, large-scale storage grids, *et al*.

Supplementary Note 15 (Supplementary Table 1): It is noted that for some diffusion-controlled materials (like $LiFePO_4$), the ionic transport through the electrode and electrolyte is rate limiting when charged/discharged at high current density.[42,43] Therefore, if one electrode is thin or with less mass loading (say, less than 0.5 mg cm^{-2}), it could exhibit better high-rate performance than the thicker one or with higher mass loading. As a result, it is recommended to deal with the comparison above correctly by considering the loading density of each electrode.

Supplementary References:

1 Shen, L. F., Uchaker, E., Zhang, X. G. & Cao, G. Z. Hydrogenated $Li_4Ti_5O_{12}$ Nanowire Arrays for High Rate Lithium Ion Batteries. *Adv. Mater.* **24**, 6502-6506 (2012).

2 Chen, S. *et al.* Self-Supported $Li_4Ti_5O_{12}$ Nanosheet Arrays for Lithium Ion Batteries with Excellent Rate Capability and Ultralong Cycle Life. *Energy Environ. Sci.* **7**, 1924-1930 (2014).

3 Haetge, J. *et al.* Ordered Large-Pore Mesoporous $Li_4Ti_5O_{12}$ Spinel Thin Film Electrodes with Nanocrystalline Framework for High Rate Rechargeable Lithium Batteries: Relationships Among Charge Storage, Electrical Conductivity, and Nanoscale Structure. *Chem. Mater.* **23**, 4384-4393 (2011).

4 Li, N. *et al.* A Self-Standing and Flexible Electrode of $Li_4Ti_5O_{12}$ Nanosheets with a N-Doped Carbon Coating for High Rate Lithium Ion Batteries. *Adv. Funct. Mater.* **23**, 5429-5435 (2013).

5 Liu, J. *et al.* Self-Supported $Li_4Ti_5O_{12}$-C Nanotube Arrays as High-Rate and Long-Life Anode Materials for Flexible Li-Ion Batteries. *Nano Lett.* **14**, 2597-2603 (2014).

6 Hasegawa, G. *et al.* Hierarchically Porous $Li_4Ti_5O_{12}$ Anode Materials for Li-and Na-Ion Batteries: Effects of Nanoarchitectural Design and Temperature Dependence of the Rate Capability. *Adv. Energy Mater.* **5**, 1400730 (2015).

7 Yu, L., Wu, H. B. & Lou, X. W. Mesoporous $Li_4Ti_5O_{12}$ Hollow Spheres with Enhanced Lithium Storage Capability. *Adv. Mater.* **25**, 2296-2300 (2013).

8 Kang, E. *et al.* Highly Improved Rate Capability for a Lithium-Ion Battery Nano-$Li_4Ti_5O_{12}$ Negative Electrode via Carbon-Coated Mesoporous Uniform Pores with a Simple Self-Assembly Method. *Adv. Funct. Mater.* **21**, 4349-4357 (2011).

9 Zhao, L. *et al.* Porous $Li_4Ti_5O_{12}$ Coated with N-Doped Carbon From Ionic Liquids for Li-Ion Batteries. *Adv. Mater.* **23**, 1385-1388 (2011).

10 Wang, C. *et al.* Combining Fast Li-Ion Battery Cycling with Large Volumetric Energy Density: Grain Boundary Induced High Electronic and Ionic Conductivity in $Li_4Ti_5O_{12}$ Spheres of Densely Packed Nanocrystallites. *Chem. Mater.* **27**, 5647-5656 (2015).

11 Wang, S. *et al.* Ti^{3+}-Free Three-Phase $Li_4Ti_5O_{12}/TiO_2$ for High-Rate Lithium Ion Batteries: Capacity and Conductivity Enhancement by Phase Boundaries. *Nano Energy* **32**, 294-301 (2017).

12 Shen, L. *et al.* $Li_4Ti_5O_{12}$ Nanoparticles Embedded in a Mesoporous Carbon Matrix as a Superior Anode Material for High Rate Lithium Ion Batteries. *Adv. Energy Mater.* **2**, 691-698 (2012).

13 Shen, L. *et al.* Advanced Energy-Storage Architectures Composed of Spinel Lithium Metal Oxide Nanocrystal On Carbon Textiles. *Adv. Energy Mater.* **3**, 1484-1489 (2013).

14 Yang, Y. *et al.* Lithium Titanate Tailored by Cathodically Induced Graphene for an Ultrafast Lithium Ion Battery. *Adv. Funct. Mater.* **24**, 4349-4356 (2014).

15 Wang, C. *et al.* A Robust Strategy for Crafting Monodisperse $Li_4Ti_5O_{12}$ Nanospheres as Superior Rate Anode for Lithium Ion Batteries. *Nano Energy* **21**, 133-144 (2016).

16 Singh, D. P., Mulder, F. M. & Wagemaker, M. Templated Spinel $Li_4Ti_5O_{12}$ Li-Ion Battery Electrodes Combining High Rates with High Energy Density. *Electrochem. Commun.* **35**, 124-127 (2013).

17 Tang, Y. *et al.* Mechanical Force-Driven Growth of Elongated Bending TiO_2-Based Nanotubular Materials for Ultrafast Rechargeable Lithium Ion Batteries. *Adv. Mater.* **26**, 6111-6118 (2014).

18 Liu, H. S. *et al.* Mesoporous TiO_2-B Microspheres with Superior Rate Performance for Lithium Ion Batteries. *Adv. Mater.* **23**, 3450-3454 (2011).

19 Liu, S. H. *et al.* Nanosheet-Constructed Porous TiO_2-B for Advanced Lithium Ion Batteries. *Adv. Mater.* **24**, 3201-3204 (2012).

20 Liu, S. H. *et al.* A Flexible TiO_2(B)-Based Battery Electrode with Superior Power Rate and Ultralong Cycle Life. *Adv. Mater.* **25**, 3462-3467 (2013).

21 Tang, Y. *et al.* Unravelling the Correlation Between the Aspect Ratio of Nanotubular Structures and their Electrochemical Performance to Achieve High-Rate and Long-Life Lithium-Ion Batteries. *Angew. Chem. Int. Ed.* **53**, 13488-13492 (2014).

22 Chen, J. *et al.* Ti^{3+} Self-Doped Dark Rutile TiO_2 Ultrafine Nanorods with Durable High-Rate Capability for Lithium-Ion Batteries. *Adv. Funct. Mater.* **25**, 6793-6801 (2015).

23 Suzuki, Y., Pavasupree, S., Yoshikawa, S. & Kawahata, R. Natural Rutile-Derived Titanate Nanofibers Prepared by Direct Hydrothermal Processing. *J. Mater. Res.* **20**, 1063-1070 (2005).

24 Leroy, S. *et al.* Influence of the Lithium Salt Nature over the Surface Film Formation on a Graphite Electrode in Li-Ion Batteries: An XPS Study. *Appl. Surf. Sci.* **253**, 4895-4905 (2007).

25 Dedryvere, R. *et al.* Characterization of Lithium Alkyl Carbonates by X-Ray Photoelectron Spectroscopy: Experimental and Theoretical Study. *J. Phys. Chem. B* **109**, 15868-15875 (2005).

26 He, Y. *et al.* Gassing in $Li_4Ti_5O_{12}$-Based Batteries and its Remedy. *Sci. Rep.* **2**, 1-9 (2012).

27 Imhof, R. In Situ Investigation of the Electrochemical Reduction of Carbonate Electrolyte Solutions at Graphite Electrodes. *J. Electrochem. Soc.* **145** 1081-1087 (1998).

28 Dedryvere, R. *et al.* Surface Film Formation on Electrodes in a $LiCoO_2$/Graphite Cell: A Step by Step XPS Study. *J. Power Sources* **174**, 462-468 (2007).

29 Wen, L. *et al.* Dual Functions of Carbon in $Li_4Ti_5O_{12}$/C Microspheres. *J. Electrochem. Soc.* **162**, A3038-A3044 (2014).

30 Yang, J., Sa, L., Akihiro, K. & Al., E. Self-Healing SEI Enables Full-Cell Cycling of a Silicon-Majority Anode with a Coulombic Efficiency Exceeding 99.9%. *Energy Environ. Sci.* **10**, 580-592 (2017).

31 Augustyn, V., Simon, P. & Dunn, B. Pseudocapacitive Oxide Materials for High-Rate Electrochemical Energy Storage. *Energy Environ. Sci.* **7**, 1597-1614 (2014).

32 Yin, H. *et al.* Influence of Morphologies and Pseudocapacitive Contributions for Charge Storage in V_2O_5 Micro/Nano-Structures. *Electrochim. Acta* **111**, 762-770 (2013).

33 Zaghib, K., Simoneau, M., Armand, M. & Gauthier, M. Electrochemical Study of $Li_4Ti_5O_{12}$ as Negative Electrode for Li-Ion Polymer Rechargeable Batteries. *J. Power Sources* **81–82**, 300-305 (1999).

34 Ho, C., Raistrick, I. D. & Huggins, R. A. Application of A-C Techniques to the Study of Lithium Diffusion in Tungsten Trioxide Thin Films. *J. Electrochem. Soc.* **127**, 343-350 (1980).

35 Zhang, D., Popov, B. N. & White, R. E. Electrochemical Investigation of $CrO_{2.65}$ Doped $LiMn_2O_4$ as a Cathode Material for Lithium-Ion Batteries. *J. Power Sources* **76**, 81-90 (1998).

36 Wang, Y. *et al.* Synthesis and Electrochemical Performance of Nano-Sized $Li_4Ti_5O_{12}$ with Double Surface Modification of Ti(iii) and Carbon. *J. Mater. Chem.* **19**, 6789-6795 (2009).

37 Yu, X. *et al.* A Size-Dependent Sodium Storage Mechanism in $Li_4Ti_5O_{12}$ Investigated by a Novel Characterization Technique Combining *in Situ* X-Ray Diffraction and Chemical Sodiation. *Nano Lett.* **13**, 4721-4727 (2013).

38 Lu, X. *et al.* New Insight into the Atomic-Scale Bulk and Surface Structure Evolution of $Li_4Ti_5O_{12}$ Anode. *J. Am. Chem. Soc.* **137**, 1581-1586 (2015).

39 Takagaki, A. *et al.* Titanium Niobate and Titanium Tantalate Nanosheets as Strong Solid Acid Catalysts. *J. Phys. Chem. B* **108**, 11549-11555 (2004).

40 Xia, T. *et al.* Hydrogenated Surface Disorder Enhances Lithium Ion Battery Performance. *Nano Energy* **2**, 826-835 (2013).

41 Li, S. *et al.* High-Rate Aluminium Yolk-Shell Nanoparticle Anode for Li-Ion Battery with Long Cycle Life and Ultrahigh Capacity. *Nat. Commun.* **6**, 7872 (2015).

42 Fongy, C. *et al.* Electronic and Ionic Wirings Versus the Insertion Reaction Contributions to the Polarization in $LiFePO_4$ Composite Electrodes. *J. Electrochem. Soc.* **157**, A1347-A1353 (2010).

43 Zhang, X., Verhallen, T. W., Labohm, F. & Wagemaker, M. Direct Observation of Li-Ion Transport in Electrodes Under Nonequilibrium Conditions Using Neutron Depth Profiling. *Adv. Energy Mater.* **5**, 1500498 (2015).
